# End-to-end robust system discovery in electrical dynamical systems using scientific machine learning

**DOI:** 10.1038/s44172-026-00689-2

**Published:** 2026-06-16

**Authors:** Jhelum Chakravorty, Nicolò Ripamonti, Tor Laneryd

**Affiliations:** 1 Hitachi Energy Research Canada, Montreal, QC Canada; 2 Hitachi Energy Research Switzerland, Baden-Dättwil, Switzerland; 3 Hitachi Energy Research Sweden, Västerås, Sweden

**Keywords:** Computational science, Thermoelectric devices and materials, Electrical and electronic engineering

## Abstract

System discovery is an important part of the power systems asset management process. In this paper, we introduce an end-to-end approach for robust system discovery for a class of electrical dynamical systems with polynomial dynamics. To that end, we provide a theoretical analysis of the problem setting and the solution approach using a particular Scientific Machine Learning method called Physics-Informed Machine Learning. We introduce model architecture and training and validation methods for deterministic as well as probabilistic approaches to predict the solution to pertinent inverse problems and propose a sampling method to make the predictions robust to data sparsity. With empirical evidence drawn from a case study of thermal modeling of electrical induction machines, we establish the merits of the proposed method and discuss the implications of this research on possible future directions. In particular, we benchmark our proposed end-to-end method with multiple deterministic and probabilistic approaches and show that it outperforms all but one baseline in terms of performance in a range of 6% to 78% reduction of validation error. The drop in performance (increase in validation error) in our end-to-end probabilistic approach compared to a Bayesian approach is compensated by a large reduction (86%) in the computation time, making our method more balanced in terms of performance and cost. The experimental results establish that our end-to-end approach outperforms the baseline methods by demonstrating consistent balancing among performance and computation cost.

## Abstract (web summary)

Jhelum Chakravorty and colleagues introduce an end-to-end approach to robust discovery of electrical dynamical systems with a class of polynomial dynamics. This research lays the ground for modelling diverse multi-physics systems with a balance in computation cost and performance.

## Introduction

Electrical and power dynamical systems, such as power transformers and shunt reactors, semiconductors, converters, etc., are guided by one or more physical laws. Examples include electromagnetic induction laws for thermal modeling and partial discharge, acoustics, Maxwell’s equations for electromagnetic field behavior, Joule heating principles, electrodynamic coupling mechanisms, electrostatic field theory, magnetohydrodynamics for cooling fluid interactions, and nonlinear material characteristics that affect core saturation. Often, the current practices for system analysis and discovery are limited to simulation-based and analytical modeling-based approaches, which are costly, can fail in the face of uncertainty, are sensitive to data sparsity, and call for repeating the analysis from scratch with any change in the underlying assumptions. In this paper, we look at the problem of system discovery of a few classes of electrical and power system dynamics. Also known as the *inverse problem*, where the *cause* of a dynamical phenomenon is predicted from the observed *effect*, this is usually a difficult problem due to a lack of ground truth data for the cause. In this paper, we use the terms system discovery and inverse solutions interchangeably.

We focus on developing an approach to predict the inverse solution from the observed effect in a robust and automated way. To that end, we invoke an emerging avenue of Machine Learning, the Scientific Machine Learning (SciML), that augments the traditional data-driven approach with system-specific domain knowledge. While SciML is quite wide in its definition (equation discovery methods, differentiable physics simulators, multi-fidelity modeling techniques, DeepONet and Fourier Neural Operators that learn mappings between function spaces, and Bayesian methods for uncertainty quantification in scientific predictions), in this paper we restrict our attention to *Physics-Informed Machine Learning* (PIML) that uses scientific knowledge encoded in the physical laws in terms of mathematical models such as Ordinary or Partial Differential Equations (ODEs/PDEs) and incorporates these laws in the training of the predictive model as additional constraints in the objective function.

Robust system discovery includes satisfactory predictions of the cause in the face of uncertainty. However, uncertainty quantification (UQ) in inverse problems is a challenging task, that addresses the variability in the data and the model to predict the cause of an event by quantifying the uncertainty in the measured effect and relating it to the uncertainty in the cause. When the measured data are noisy or the mathematical model for the underlying dynamics is imperfect, it becomes important to quantify the uncertainties inherent in any inferences derived from the solutions of the inverse problems, which also helps identify and regularize any *ill-posedness* in the solution of the inverse problem, i.e., conditions violating existence, uniqueness, and continuous dependence of solutions on data. For the inverse problems relating to condition monitoring in power devices, this is instrumental for understanding the uncertainties in the observed effect in inaccessible components by measuring the values of the accessible interconnected parts, which makes the prediction accurate as well as robust to varying reliability of the data quality. Furthermore, for some electrical dynamical system discovery applications, the data available is sparse because of poor accessibility of certain regions for data procurement. It is important that the predictions of system dynamics is robust with data sparsity.

This research can benefit electrical condition monitoring applications by providing a robust framework for inverse problem solutions. In electrical acoustics, this approach could help identify partial discharge locations from limited acoustic emissions data. For high-voltage equipment monitoring, the methodology enables more accurate source localization from electromagnetic signals, while transformer vibration analysis could leverage this framework to identify mechanical or core issues from external measurements. The methodology extends beyond electrical systems to various industrial applications. For industrial process monitoring, it could identify anomalies in chemical reactions without direct access to reaction chambers. In structural health monitoring, this approach could locate weaknesses from limited sensor data, while in semiconductor manufacturing, it could identify process variations from indirect measurements. In each case, the robust inverse solution methodology and uncertainty quantification provide valuable decision support, reducing downtime, preventing catastrophic failures, and optimizing maintenance intervals across critical infrastructure. This framework’s ability to handle sparse and noisy data while providing confidence metrics improves decision-making in predictive maintenance scenarios. The developed inverse solution methodology and uncertainty quantification enhance manufacturing processes by enabling data-driven predictive maintenance of electrical systems, reducing downtime, and optimizing maintenance intervals across critical manufacturing infrastructure. The end-to-end approach proposed in this paper lays the ground for enhanced condition monitoring, root cause analysis, and system discovery in the face of uncertainty through detailed analysis and empirical evidence. This methodology can be applied to dynamical systems where the underlying physics is known or can be modeled, and the data availability is limited and may be noisy, sparse, or inaccessible. The applicability of the proposed method in diverse realistic scenarios is primarily affected by the balance between performance and computation cost that this approach offers, which is established through experimental results in the paper. Some example applications that can benefit from the proposed unified framework include, but are not limited to, condition monitoring in diverse electrical and power systems assets^1^, model order reduction for manufacturing^2^ and system discovery^3,4^.

Scientific Machine Learning (SciML) encompasses a diverse range of methods beyond Physics-Informed Machine Learning. Equation discovery methods like SINDy^5^ (Sparse Identification of Nonlinear Dynamics) extract governing equations directly from data without prior knowledge of system structure. Differentiable physics simulators^6^ enable end-to-end optimization by embedding physical constraints in differentiable computational graphs. Operator learning approaches such as DeepONet^7^ and Fourier Neural Operators^8^ learn mappings between function spaces, effectively capturing solution operators for families of PDEs rather than solving individual instances. Bayesian methods for scientific computing provide robust uncertainty quantification in predictions while incorporating domain knowledge through carefully designed priors. PIML, a type of SciML method that integrates theoretical knowledge and observational data, has shown remarkable success in handling inverse problems related to Partial Differential Equations (PDEs)^9^. In situations where only parts of the system description, i.e., initial or boundary conditions and coefficients, are available, PIML excels at estimating missing inputs through insights derived from collected data^10^. Unlike traditional approaches^11^, PIML can work with limited, noisy data by integrating domain knowledge with mathematical models^12^. Consequently, PIML solutions often claim increased accuracy, faster training, comprehensibility, and generalization, thanks to the interplay between machine learning capabilities and physical laws. Physics-Informed Neural Network (PINN) is a way to implement PIML by integrating a Deep Neural Network (DNN) along with a mathematical model such as an ODE/PDE.

Rules for measurements of power losses in transformers is described in international standards and guides^13,14^, including determination of uncertainties^15^. A body of work exists that reviews of transformer losses^16,17^, combines experiments and computation^18^ and describes the use of thermal cameras to estimate losses^19–22^. The method presented in ref. ^18^ estimates parameters to compute the temperature distribution on the transformer top using multi-objective optimization. The work in ref. ^20^ studies on the reliability of thermal measurements by comparing the core loss values calculated using thermal measurements with the same directly measured using magnetic methods. The work in ref. ^21^ focuses on solving Maxwell’s equations using measured infrared images of temperature distribution in a physics simulator to compute core losses, where access to measure ground truths is also available. The authors of ref. ^19^ proposes a method to estimate losses in a high-frequency planar transformer by first calibrating the measurement equipment (thermal camera) by heating transformer components using a DC power supply, and then estimating power losses in the tested transformer under predefined loads using the heating curves obtained during the calibration step. These estimates are compared with ground truth calculated using established theoretical electrical relationships. The approach of ref. ^22^ uses thermographic images to calculate total losses in dry-type air-core reactors using the calorimetric method. In contrast with these related works for estimating power loss in a device, we propose a nonlinear predictive tool (PIML) that can estimate directly from measured temperature distributions, can create an estimate of the heat loss without any direct comparison with ground-truth (hence being applicable even in the absence of ground truth), and is much faster than the physics simulators to predict the heat losses during inference.

Moreover, our approach computes the predictive model’s confidence in its estimation through an uncertainty quantification method, which adds to the model’s interpretability. Uncertainty quantification is a research area occurring in mathematical modeling, probability, statistics and machine learning. In the probabilistic setting, Bayesian approaches such as Bayesian networks^23^, quantify the uncertainties in the predictions. However, Bayesian approaches are usually computationally expensive, e.g., with Monte Carlo search^24^ or training Bayesian Networks^25^. In contrast, our approach for quantifying uncertainty provides a computationally cheaper way to estimate uncertainty with Gaussian parameterization.

The rest of the paper is organized as follows. In Methods section we discuss the main aspects of the overall methods: a unified framework for the end-to-end automated and robust system discovery that formulates the inverse problem in both deterministic as well as probabilistic setting, the application of PIML in predicting local heat loss distribution in electromagnetic induction devices with the details on the thermal modeling and data procurement and processing and the methodology for using PIML for this use case including the PINN model architectures and training and validation, introduction of active sampling for improving performance, analysis of model’s robustness with data sparsity and description of an indirect validation method for the PINN models in the absence of ground truth data for heat loss. To that end, we present a theoretical analysis of the uncertainty quantification in the probabilistic setting and provide relevant mathematical details in Uncertainty Quantification in Polynomial Dynamics and Singularity Avoidance in Inverse Problems section of the supplementary document. The experimental results are discussed in the Results and discussion section. In this section, we show empirical results in the deterministic and probabilistic settings and for active sampling and robustness analysis, compare the results with a range of baseline methods, and discuss the implications of these results. Additionally, we benchmark the propose the end-to-end approach with 1-D Burgers equation and 2-D Compressible Navier Stokes dynamics in the Benchmarking of UQ in Nonlinear Dynamics with Burgers Equation and Benchmarking of UQ in 2-D Nonlinear Dynamics with Compressible Navier Stokes Equations sections of the supplementary document. The Conclusion section summarizes the highlights of the paper and discusses the future scope of this research. Data Availability Statement and Code Availability Statement sections provide statements for data and code availability, and the author information is given in the Author contributions section. In the Competing interests section, competing interests are declared.

## Methods

We use PINNs as a predictive tool for solving the heat loss localization inverse problem in power devices, using limited medium-quality data and partial knowledge of heat diffusion dynamics. We introduce uncertainty quantification (UQ) for PINN models for electrical dynamical systems. Throughout the paper, we present different training losses balancing computation cost (model size and convergence time) against prediction accuracy, while improving model performance and interpretability.

Given the absence of ground truth data, we develop an indirect validation method for trained PINN models. A key aspect of our contribution is the development of a validation strategy in case of real-world scenarios where direct ground truth for the inferred quantities is inaccessible. We acknowledge that our proposed indirect validation method carries the assumption that the physical model, in this case, the heat diffusion equation, is a sufficiently accurate representation of the true thermal dynamics. This can introduce a bias, as the validation primarily confirms the PINN’s success in learning to invert the assumed physical laws, rather than the laws of the real-world system. This challenge, known as model discrepancy, is a fundamental and well-known issue in the field of inverse problems, where simplified models are a practical necessity for computational tractability. Our contribution, therefore, lies in providing a complete, computationally efficient framework that delivers valuable insights under these practical constraints. While this emerging research direction offers limited baseline comparisons, our comprehensive analysis of the case study involving inverse heat diffusion problem establishes foundations for applying PIML and UQ to wider ranges of dynamical systems. We acknowledge that explicitly addressing model discrepancy could be a natural extension of the proposed work.

### End-to-end automated and robust system discovery for electrical dynamical systems

We recognize that an end-to-end automated system discovery approach for electrical and power dynamical systems should leverage information from measured observations (effects) and complete or partial knowledge of the underlying system dynamics and provide predictions of the cause with satisfactory performance (e.g., accuracy) even in the presence of limited, unreliable, or moderate-quality data. Moreover, analyses such as uncertainty quantification in effect and predicted cause and robustness analysis with data sparsity can provide useful insights towards explainable predictions that may help human experts interpret the outputs and take actions accordingly.

To that end, we propose a unified framework for end-to-end (E2E) and modular approach in deterministic as well as probabilistic settings for finding robust inverse solutions for a class of dynamical systems arising in electrical and power engineering. The main modules of this holistic approach are as follows:

- **Data collection and processing**: Procure or generate data, e.g., point data using thermocouples and images using a thermal camera. Include necessary processing for using in PIML models.
- **PIML-based inverse solutions**: Use PIML to find point-wise (deterministic) or computationally efficient probabilistic prediction of cause dynamics using observed effect.
- **Robustness analysis of prediction with data sparsity**: We propose an *Active Learning* based sampling method to leverage UQ signals to sample a few relevant data points that can result in satisfactory performance.

**Fig. 1:**
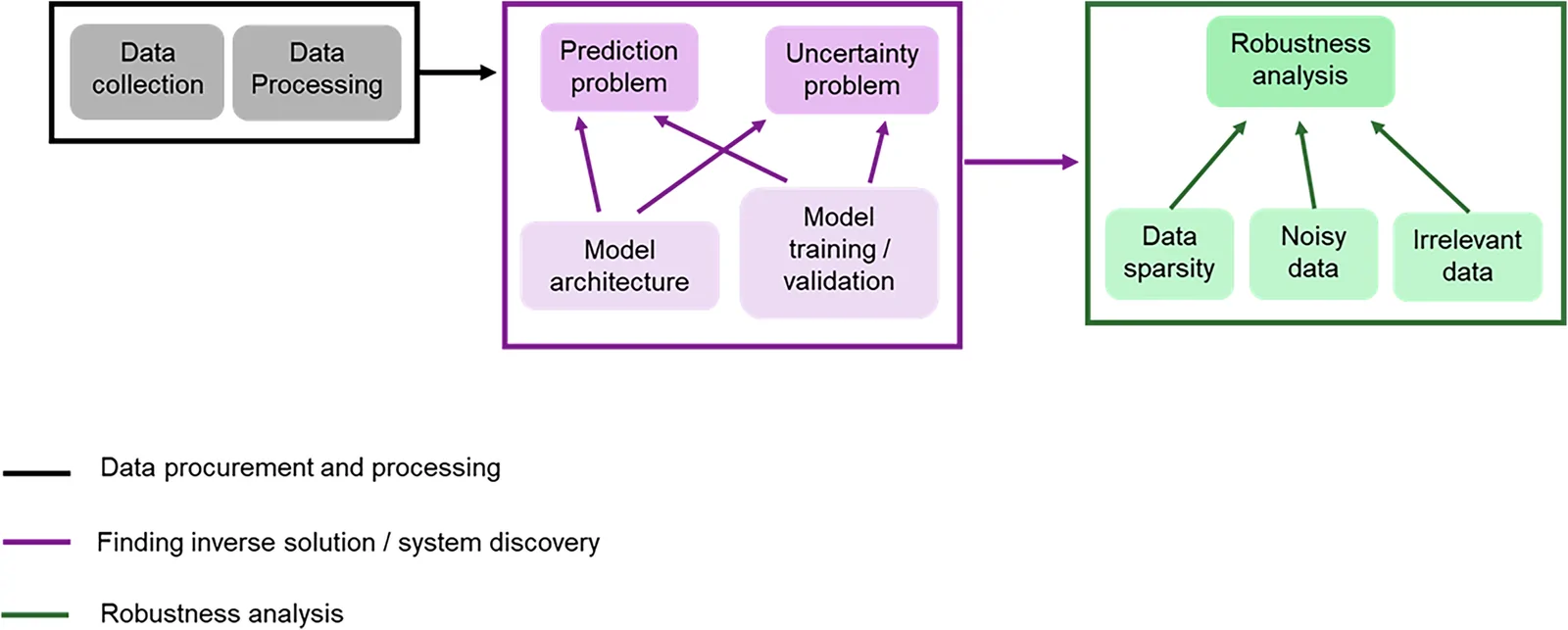
Block diagram illustrating the main modules for an end-to-end process for automated and robust system discovery proposed in this paper. The first block (in gray) represents the data procurement and processing, followed by the second block (in purple) that depicts the method for finding inverse solution by selecting appropriate model architecture, training and validation for prediction as well as uncertainty quantification problem. The third block (in green) shows the robustness analysis method to account for sparse, noisy or irrelevant data.

The end-to-end process is illustrated in Fig. 1.

Whereas the data procurement and processing may vary widely depending on the modality of data required, e.g., thermal images or point measurements by thermocouples for system discovery for a thermal modeling problem, it is a very important step for successfully solving the inverse problem using SciML methods. Preparing data to be used as the input to the predictive models involves processing such as denoising, edge-detection, normalization etc. We discuss the data collection and pre-processing for a given use case in Application of PIML for predicting local heat loss distribution in electromagnetic induction devices. In the rest of this section, we develop the problem formulation and its associated theory for robustly solving inverse problems.

#### Problem formulation

We describe both the prediction and the UQ problems in the electrical induction machines and introduce the PINN setup for solving the inverse problem.

##### Prediction problem

The problem of system discovery for a class of electrical and power dynamical systems essentially boils down to predicting the cause ${{{\mathcal{C}}}}$, which can be a variable or a function, from the observed effect ${{{\mathcal{E}}}}$, which is often a function (e.g., spatiotemporal) connected to the cause through the underlying physics *F* as follows:

1 $$F[{{{\mathcal{E}}}};{{{\mathcal{C}}}}]=0.$$

Let *f*_*θ*_(*x*, *t*) be the neural network approximating the cause ${{{\mathcal{C}}}}$ given in (1). While traditional deep learning^26^ minimizes the mismatch between network output and target values by fine-tuning the parameters *θ*, inverse problems present a unique challenge: the target cannot be directly measured. Instead, PIML compensates for this lack of ground truth by learning to predict the cause through the physical dynamics that connect them to measurable effect distributions.

To ensure the accuracy of the prediction, it is necessary that the neural network satisfies the physics governed by the PDE. This result is satisfied in a weak sense by restricting the training of the network on a set of collocation points ${\Omega }_{{{{\bf{x}}}}}=\{{{{{\bf{x}}}}}_{1},{{{{\bf{x}}}}}_{2},\ldots ,{{{{\bf{x}}}}}_{| {\Omega }_{{{{\bf{x}}}}}| }\}$ and ${\Omega }_{{{{\bf{t}}}}}=\{{t}_{1},{t}_{2},\ldots ,{t}_{| {\Omega }_{{{{\bf{t}}}}}| }\}$, with *Ω* ≔ *Ω*_**x**_ × *Ω*_*t*_, that resembles the space-time discretization of traditional computational methods. These points can be drawn from a distribution, or, in an experimental setting, they are the sampled signals from sensors or camera measurements. We therefore assume that measurements are available for these points in the form of time series and, for the sake of notation, we denote both the measured values of the effect and the values that appear in equation (1) with ${{{\mathcal{E}}}}$, thus ignoring physical modeling errors.

The error ${{{{\mathcal{L}}}}}_{2}$ between the outputs of the neural networks ($\widehat{{{{\mathcal{C}}}}}$ and $\widehat{{{{\mathcal{E}}}}}$) and the measured observations and the discrepancy ${{{{\mathcal{L}}}}}_{1}$ with the solution of the physical model (1) are measured by the weighted sum of the *L*_2_ norm (denoted by ∥ ⋅ ∥_2_) of the PDE residual and the temperature mismatch at the collocation points (adopted from Eq. (2)^27^, reproduced with permission from CIGRE):

2 $${{{\mathcal{L}}}}(\theta ;\Omega )={\omega }_{1}{{{{\mathcal{L}}}}}_{1}(\theta ;\Omega )+{\omega }_{2}{{{{\mathcal{L}}}}}_{2}(\theta ;\Omega )$$

with *ω*_1_ and *ω*_2_ as optimization weights and ${{{{\mathcal{L}}}}}_{1}$ and ${{{{\mathcal{L}}}}}_{2}$ are expressed as

3 $${{{{\mathcal{L}}}}}_{1}(\theta ;\Omega ):=\frac{1}{| {\Omega }_{{{{\bf{x}}}}}| | {\Omega }_{t}| }{\sum}_{{{{\bf{x}}}}\in {\Omega }_{{{{\bf{x}}}}},t\in {\Omega }_{t}}\parallel F[\widehat{{{{\mathcal{E}}}}};\widehat{{{{\mathcal{C}}}}}]{\parallel }_{2}^{2},$$

and

4 $${{{{\mathcal{L}}}}}_{2}(\theta ;\Omega ):=\frac{1}{| {\Omega }_{{{{\bf{x}}}}}| | {\Omega }_{t}| }{\sum}_{{{{\bf{x}}}}\in {\Omega }_{{{{\bf{x}}}}},t\in {\Omega }_{t}}\parallel \widehat{{{{\mathcal{E}}}}}-{{{\mathcal{E}}}}{\parallel }_{2}^{2}.$$

The PDE residual ${{{{\mathcal{L}}}}}_{1}$ in (3) is computed using automatic differentiation^28^, a graph-based differentiation method to compute derivatives with working precision, provided in any popular machine learning library.

The trainable parameters *θ* are optimized by minimizing the loss function ${{{\mathcal{L}}}}$ given by (2) using the first-order stochastic gradient descent based method ADAM^29^.

##### Uncertainty quantification problem

In this section, inspired from ref. ^30^, we extend the deterministic PINN for DNNs, that is computationally cheaper than the Bayesian approaches.

In this probabilistic setting, we consider that the variable of interest is probabilistic with a Gaussian distribution. We train the PINN model to estimate the mean and variance of the Gaussian distribution. For ease of exposition, we henceforth call the PINN model equipped with UQ the *Pro-PINN*. To establish this UQ for inverse solutions, we first develop a theory of quantifying uncertainty in cause and effect in a class of system dynamics and then elaborate the method for a specific class of inverse problem, the thermal modeling of electromagnetic power devices, in the later sections of the paper.

In this work, we provide a detailed analysis for formulating a class of inverse problems and develop mathematical expressions for formulating UQ in such problems. We establish the expressions for the inverse solutions in such settings, address the challenges that can make the inverse problems ill-posed, and discuss workarounds to find the inverse solutions. In this section, we summarize the steps towards formulating the theory of uncertainty quantification in cause and effect in polynomial dynamics. For the ease of readability, the analytical details of the formulation of polynomial dynamics and the uncertainty quantification in such dynamics are given in Uncertainty Quantification in Polynomial Dynamics and Singularity Avoidance in Inverse Problems section of the supplementary document.

Our goal is to understand how the uncertainty in our measurements ${{{\mathcal{E}}}}$ translates into uncertainty in the approximation of the cause ${{{\mathcal{C}}}}$. The derivation follows four main steps:

1. We first look at the physics equation linking the cause and effect. We show that if the cause is simply added to the equation, it’s easy to solve for. But if it multiplies other terms, we can run into ‘division by zero’ problems (singularities) that make the solution unstable.
2. We assume the uncertainty in our measurement ${{{\mathcal{E}}}}$ follows a standard Gaussian distribution.
3. We then mathematically trace how this uncertainty gets distorted as it passes through the physics equation. We find that simple parts (like derivatives) are still distributed as Gaussian, but polynomials (like ${{{{\mathcal{E}}}}}^{2}$ or ${{{{\mathcal{E}}}}}^{3}$) do not follow a Gaussian distribution.
4. Finally, we propose two practical solutions: (a) using special methods to handle the non-Gaussian distributions from Step 3, and (b) a singularity avoidance technique that finds the unstable ‘division by zero’ data points from Step 1 and reduces or removes their relevance in the training.

### Application of PIML for predicting local heat loss distribution in electromagnetic induction devices

In this section, we develop the end-to-end approach to the robust solution for the inverse problem in a case study of thermal diffusion problems arising from local heat losses in electrical induction machines such as power transformers and shunt reactors. The heat losses in power devices represent both an economic loss and an unnecessary emission of greenhouse gases. Raising the temperature level reduces the remaining useful life (RUL) through accelerated thermal aging and may require an additional cooling system. An important first step in reducing heat losses is to localize where in the power device they occur.

For power transformers, the total losses are the difference between the input and output power, and their measurement is relatively straightforward. The distribution of heat losses is more difficult to obtain. Instead, measuring the temperature distribution and calculating the heat losses that would cause this thermal behavior is an example of an inverse problem, where one estimates the cause of an event by observing the effect of the same. In this paper, we present an approach to solve this inverse problem using PIML.

#### Thermal modeling of electric induction devices

In this section, we formulate the inverse problem of predicting local heat generation due to heat diffusion processes on the tank walls of the electrical induction devices, such as power transformers and shunt reactors, and provide thermal diffusion dynamical modeling for electrical induction devices.

**Fig. 2:**
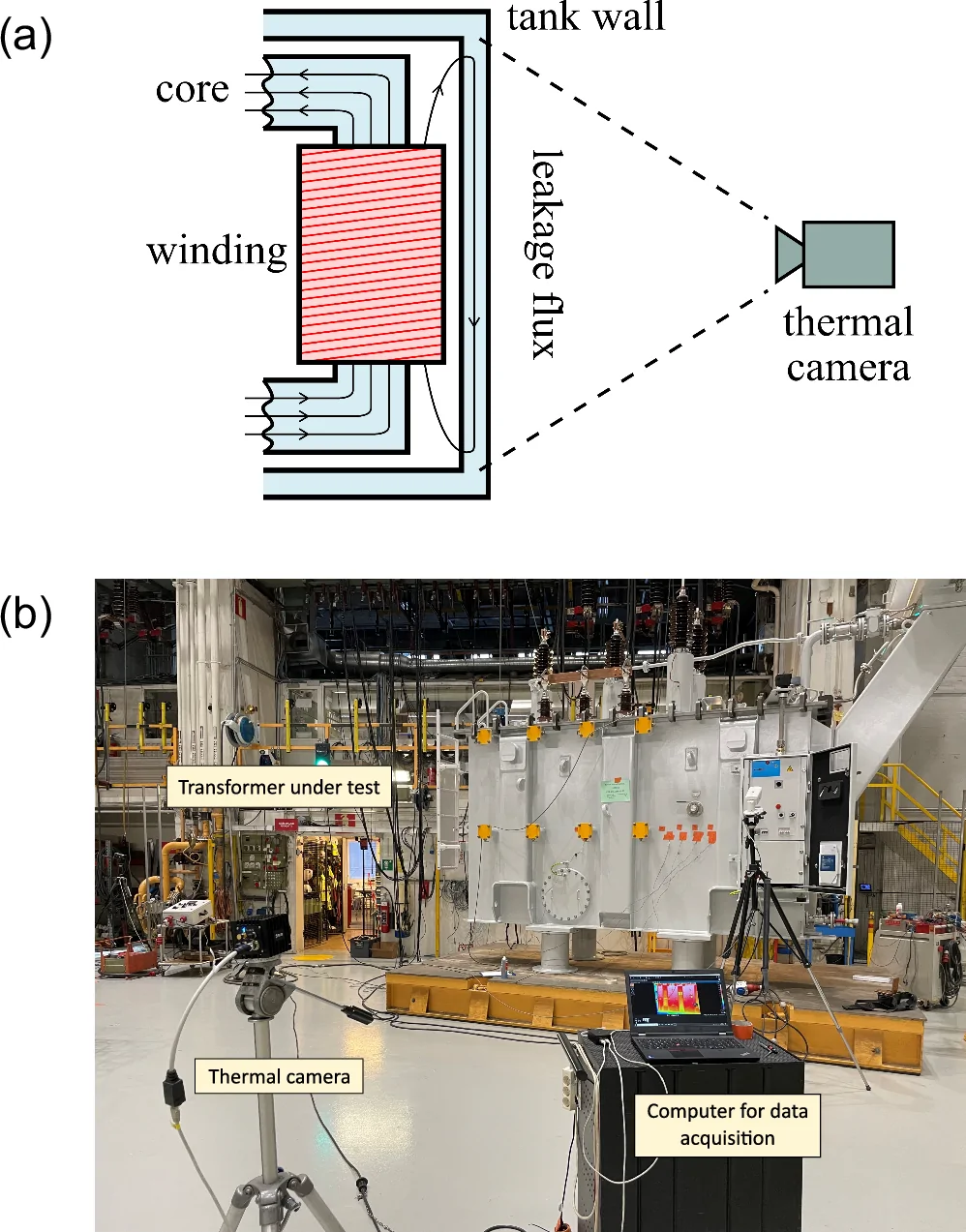
Prediction of local heat loss distribution in electrical induction machines. **a** Schematic diagram for the path of leakage flux in power transformers and shunt reactors. **b** Experimental set-up for measuring temperature distributions on tank-walls of devices in a factory (adopted from Fig. 3^27^, reproduced with permission from CIGRE).

While heat transfer occurs through conduction, convection, and radiation both within and from the power device, in this work, we focus on electric power losses in the transformer or shunt reactor tank-walls. Given that the tank-wall is relatively thin, we simplify the analysis to a planar surface with oil and air on opposing sides, neglecting transversal temperature variations:

5 $${c}_{p}\rho \frac{\partial T}{\partial t}-k{\nabla }^{2}T=q,$$

where the material properties of the tank-wall are denoted by *c*_*p*_ for the specific heat capacity at constant pressure and *ρ* is the density of the tank-wall material and *k* is thermal conductivity of the material of the device wall. The total heat generation *q* is the heat loss density *P* multiplied by tank-wall thickness *d* and then subtracted by the cooling towards the surrounding media

6 $$q=Pd-{h}_{oil}A(T-{T}_{oil})-{h}_{air}A(T-{T}_{air}),$$

where *h*_*oil*_ and *h*_*air*_ are heat transfer coefficients to the oil and air, respectively, *T*_*oil*_ and *T*_*air*_ are the temperatures of the oil and air, and *A* is the area of the cooling surface. A similar model was presented in ref. ^31^. The temperature *T* of the tank-wall can be acquired by a thermal camera as shown in Fig. 2a. Given sufficient time before energizing, the device reaches thermal equilibrium with its surroundings, where *T*_*oil*_ and *T*_*air*_ are equal to the factory’s ambient temperature *T*_*∞*_. For short measurement durations, these temperatures remain constant. To improve neural network convergence, we non-dimensionalize the governing equation to scale solutions between 0 and 1. So, a change of scaling is applied to Eq. (5) to obtain the non-dimensional heat equation (adapted from the derivation in Section 2.2^27^, reproduced with permission from CIGRE)

7 $$\frac{\partial \widehat{T}}{\partial \widehat{t}}-D{\nabla }^{2}\widehat{T}-\widehat{q}=0,$$

where *D* is the normalized thermal conductivity coefficient given by (adapted from the derivation in Section 2.2^27^, reproduced with permission from CIGRE):

8 $$D=\frac{4k\Delta {T}_{ref}}{{L}^{2}{P}_{ref}},$$

where *ΔT*_*ref*_ ≔ *T*_*max*_ − *T*_*air*_ is the difference between the maximum tank wall temperature and the room temperature during the hear run test, *P*_*ref*_ is the reference heat generation rate required to keep the wall at maximum temperature during steady state, neglecting heat diffusion in the wall and *L* is the length of tank wall. to obtain Eq. (7) involves transformations $\widehat{x}=2x/L$, $\widehat{y}=2y/L$ and $\widehat{T}=(T-{T}_{air})/\Delta {T}_{ref}$. The boundary value problem is completed by the Dirichlet boundary constraint, $\widehat{T}{| }_{\partial \Omega }={\widehat{T}}_{\partial \Omega }$,

9 $$\widehat{T}{| }_{\partial \Omega }={\widehat{T}}_{\partial \Omega },$$

where we assume that the boundary value (defined on boundary ∂*Ω*) can be time-dependent and can be measured using, for example, a thermal camera. Note that the heat diffusion problem is a specific instance of the formulation in Eq. (1) where *F* is given by Eq. (7) with the cause ${{{\mathcal{C}}}}$ being the heat $\widehat{q}$ and the effect ${{{\mathcal{E}}}}$ being the temperature $\widehat{T}$. For ease of reference, we will drop the $\widehat{\cdot }$ notation from the normalized variables in the rest of the paper.

#### Data acquisition and processing

Measurement data are gathered through tests conducted in a factory test room on two devices: a 90 MVA power transformer (3-minute test) and a 120 MVAr shunt reactor (10-minute test) under full voltage starting from thermal equilibrium. These short durations ensure uniform, constant temperature in the cooling media. Measurements use a FLIR A70 thermal camera (Teledyne FLIR) with 29^∘^ field of view, capturing thermal images at frequencies between 1−30*Hz* with 640 × 480 resolution. The accuracy of the camera is ±2^∘^*C* ( ± 3. 6^∘^*F*) or ±2% of reading, for ambient temperature 15 ^∘^*C*−35 ^∘^*C* (59^∘^*F* − 95^∘^*F*) and object temperature above 0 ^∘^*C* (32^∘^*F*). The sensitivity of the thermal camera, given by Noise Equivalent Temperature Difference (NETD) is  < 35*mK* (milliKelvin). The test setup for the 90 MVA transformer is depicted in Fig. 2b^27^.

**Fig. 3:**
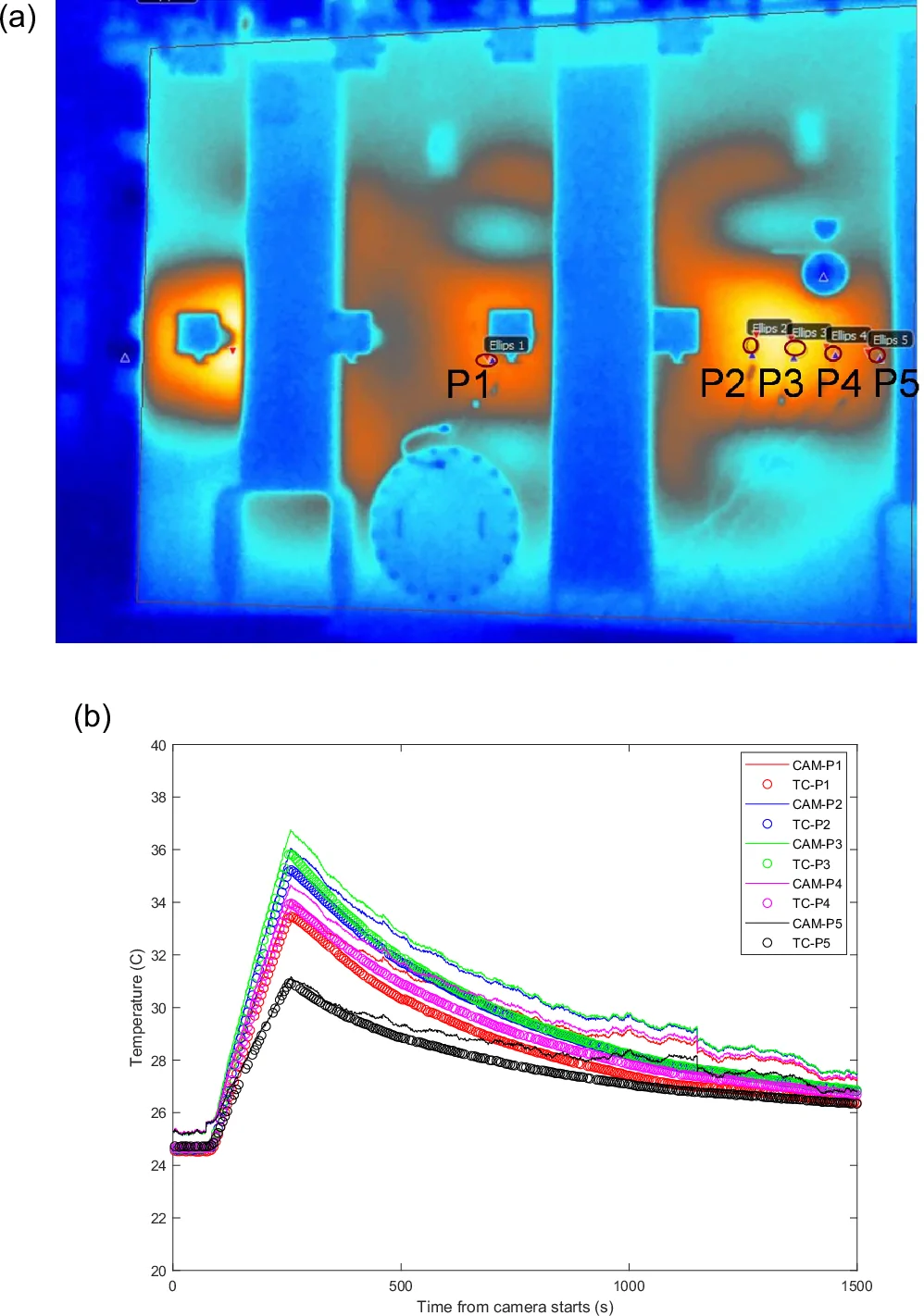
Validation of the use of a thermal camera for data measurement by comparison with measurements with thermocouples. **a** Placements of thermocouples on the tank-wall. **b** Comparison of thermal camera measurements with those given by thermocouples.

Since the measurements of thermal cameras, although spatially dense, can have measurement noise, it is important to validate the use of thermal cameras as a tool for measuring data. Since thermocouples are used widely for data measurements and they provide sufficiently precise point-wise measurements, we use the thermocouple data from given points on the tank wall as reference values and compare them with the thermal camera measurements to calibrate the latter as a reliable tool for data procurement. The comparison results are shown in Fig. 3. Figure 3a shows the placements of the thermocouples, and Fig. 3b shows a comparison of thermal camera measurements with those given by thermocouples. Figure 3b shows a good alignment of the thermal camera measurements with the thermocouple, which validates the use of the thermal camera to collect measurement data for the factory test.

Additional processing identifies unreliable pixels using *edge-detection* techniques^32^, replacing them through *interpolation* from surrounding pixel values. We hereby note that this case study focuses on capturing 2-dimensional (2-D) thermal data from the device wall to solve the inverse problem. However, this method can be extended to collect 3-D thermal data, in case of inverse problems involving volumetric components, using 3-D point cloud data created by a thermal camera along with a LiDAR scanner or another 3-D imaging device^33–35^. PIML approach can also be employed to improve the mathematical modeling of the underlying physics in a 3-D setting^36^ or for a longer time interval^37^.

### Methodology for PIML

This section describes the main components of the methodology for the application described in the Application of PIML for predicting local heat loss distribution in electromagnetic induction devices section. In particular, we discuss the architectures of PIML predictive models for estimating dynamics and heat distribution and their training and validation approaches with and without uncertainty quantification. PIML solves inverse problems directly from measurements, eliminating physics simulators to solve underlying dynamics, offering a computationally cheaper and faster parametric nonlinear model. This technology has limited equivalent methods for comparison in industrial applications.

Although focused on heat loss localization inverse problems^21^, PINN-based approaches show notable computational benefits for forward problems as well. We also test the method with synthetic examples of Gaussian and Sinc functions, where PINNs achieve prediction times two orders of magnitude faster than traditional physics simulators while maintaining accuracy in heat loss estimation.

#### PINN model architectures

In this section, we investigate two PIML model variants: deterministic PINN models providing point-wise estimates of local heat loss, and probabilistic PINNs (Pro-PINNs) that output both estimates and confidence intervals around these local heat loss values.

##### Deterministic PINN model

**Fig. 4:**
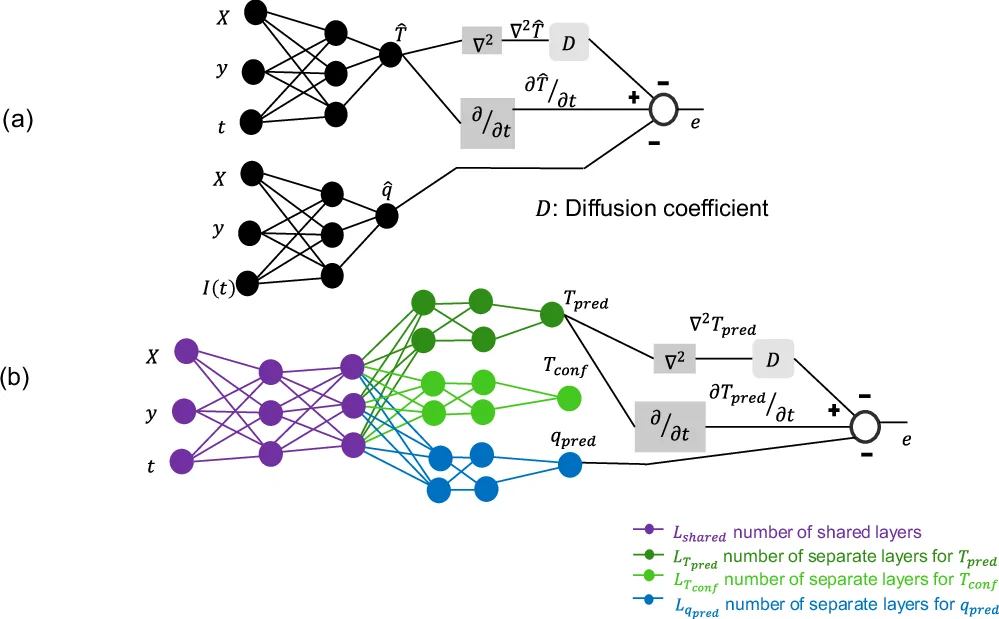
Two variants of PINN architecture for heat diffusion problem. **a** Separate architecture for deterministic PINN (adopted from Fig. 6a^27^, reproduced with permission from CIGRE). **b** Shared architecture for semi Pro-PINN.

The PINN model for the inverse problem (Fig. 4a^27^) estimates heat loss (cause) by learning the heat diffusion PDE and temperature measurements (effect). Here, *e* denotes the residual of the heat diffusion dynamics (ideally *e* = 0), (*X*, *Y*) and *t* denote the 2-D spatial coordinates and time samples, respectively and *I*(*t*) denotes the loading current, $\widehat{q}$ and $\widehat{T}$ denote the predicted local heat loss and the temperature distributions, respectively. In the ‘separate architecture’ variant (Fig. 4a^27^), all variables playing a role in the heat diffusion PDE are realized by separate DNN architectures. To reduce computational cost, we propose an alternative ‘shared architecture’ that shares parameters between layers. Empirical results in the Results and discussion section show that the shared architecture achieves faster convergence compared to the separate architecture, though with some loss in accuracy.

##### Probabilistic PINN model (Pro-PINN)

In the deterministic PINN, as in ref. ^11^, the dynamics of the physical system are predicted, and the point-wise estimates of the variables of interest are given by the PINN model. In contrast, to facilitate UQ in PINN, we assume that the variables of interest are probabilistic and the PINN model estimates the parameters of a parameterized probability density. In particular, for the thermal diffusion model, we assume that the temperature and load loss (heat) are normally distributed, and the mean and variance of the Gaussian distribution are estimated using a PINN model. In particular, the predicted temperature $\widehat{T}$ is assumed to have a Gaussian distribution $\widehat{T}(x,y,t) \sim {{{\mathcal{N}}}}({T}_{pred}(x,y,t),{T}_{conf}^{2}(x,y,t))$ with mean *T*_*pred*_ and standard deviation *T*_*conf*_. Figure 4b shows the shared architecture where the probabilistic structure is applied to *T* (semi Pro-PINN), so that $\widehat{q}(x,y,t)={q}_{pred}(x,y,t)$. Similarly, one may also impose the probabilistic structure on both *T* and *q* (full Pro-PINN). As for its deterministic counterpart, Pro-PINN too can have two variants of the architecture–separated and shared.

#### Training and validation of PINN models

##### Training and validation of deterministic PINN models

- **Myopic training**. Eq. (2) describes the training loss for the deterministic PINN, where the loss comprises components that take into account the model’s prediction of the variables and the PDE residual at that instant. We call this training method *myopic* since it only uses instantaneous information to drive the training. This training is computationally cheap and can achieve sufficient accuracy for some dynamical systems, where information from distant past does not play a crucial role in model’s prediction.
- **History-aware training**. Previous studies^38^ have shown that many of the critical issues regarding the accuracy of PINNs that may arise from PDE-based regularization and make the problem ill-conditioned can be resolved by optimizing the objective function or the training regime over time and keeping the network structure unchanged. Prediction accuracy of the dynamics can be affected by the estimation error in the past, since in a dynamical system, the estimation error propagates through the underlying PDE and affects convergence speed. If the estimation errors in earlier training episodes are considerably high, it impacts the prediction accuracy of later episodes, which in turn affects convergence speed. One way of forcing the impact of past errors to be low is to penalize past high errors by introducing exponential weights to temporal losses, with weights being a function of all past losses^39^, where the past losses are penalized and PINN loss is revised (2) as follows:
  10 $${{{\mathcal{L}}}}(\theta ;\Omega )=\frac{1}{| {\Omega }_{{{{\bf{x}}}}}| | {\Omega }_{t}| }{\sum}_{{{{\bf{x}}}}\in {\Omega }_{{{{\bf{x}}}}}}{\sum}_{t\in {\Omega }_{t}}\omega ({{{\bf{x}}}},t){{{{\mathcal{L}}}}}_{PINN}({{{\bf{x}}}},t),$$
  where ${{{{\mathcal{L}}}}}_{PINN}({{{\bf{x}}}},t)$ is the sum of the PDE residual and data mismatch and
  11 $$\omega ({{{\bf{x}}}},t)=\exp \left(-\epsilon {\sum}_{\tau \in {\Omega }_{t-1}}{{{{\mathcal{L}}}}}_{PINN}({{{\bf{x}}}},\tau )\right).$$
  with ${\Omega }_{{{{\bf{t}}}}-1}=\{{t}_{1},{t}_{2},\ldots ,{t}_{| {\Omega }_{{{{\bf{t}}}}}| -1}\}$. The approach in ref. ^39^ has shown to improve results for *forward* problems in dynamical systems where information flows from known initial conditions and the cause to predict effects. We apply this method to inverse problems, predicting local heat loss distribution (cause) from observed temperature samples (effect), often with incomplete model information. However, since the causal training given by training loss (10), (11) accounts for *all* past losses, this becomes computationally heavy with long histories. Moreover, examining the entire history may be unnecessary since the recent past captures the more significant propagated errors through the dynamics, and intermediate information from temperature time series is available beyond just initial condition propagation. So, we relax this penalty as is shown below:
  12 $$\omega ({{{\bf{x}}}},t)= \exp \left(-\epsilon {\sum}_{\tau \in {\Omega }_{t-n:t-1}}{{{{\mathcal{L}}}}}_{PINN}({{{\bf{x}}}},\tau )\left|\frac{\partial \tau }{\partial {{{{\mathcal{L}}}}}_{PINN}({{{\bf{x}}}},\tau )}\right|\right),$$
  where ${\Omega }_{t-n:t-1}:=\{{t}_{| {\Omega }_{{{{\bf{t}}}}}| -n}\ldots ,{t}_{| {\Omega }_{{{{\bf{t}}}}}| -1}\}$. In this approach of *history-aware* training, the gradient information captures the significant change in the loss since the last instant, and multiplying with the inverse gradient will make the weights high for the instants where the change in training loss is high, forcing the training process to lower the losses at those instants further. The length of the sliding window *n* is a hyper-parameter and can be optimized for a given application.

##### Training and validation of probabilistic PINN models

In a probabilistic setting, the training loss needs to be modified from its deterministic counterpart to accommodate the estimation of mean and variance separately. For the heat diffusion inverse problem, the training loss *L*_*total*_ for the Pro-PINN model, obtained by modifying (2) to plug-in $\widehat{T} \sim {{{\mathcal{N}}}}({T}_{pred},{T}_{conf}^{2})$ and $\widehat{q} \sim {{{\mathcal{N}}}}({q}_{pred},{q}_{conf}^{2})$, is the weighted average of the probabilistic loss *L*_*prob*_ (following^30^) and the PDE residual *L*_*PDE*_ for heat diffusion as follows:

13 $${L}_{prob} := 	\frac{{({T}_{pred}-T)}^{2}}{{T}_{conf}^{2}}+\log (2\pi {T}_{conf}^{2})\\ {L}_{PDE} := 	\frac{\partial }{\partial t}\widehat{T}(x,y,t)+D\frac{{\partial }^{2}}{\partial {x}^{2}}\widehat{T}(x,y,t)-\widehat{q}(x,y,t).$$

Note that the presence of ${T}_{conf}^{2}$ in the denominator of the first term of *L*_*prob*_ and in the second term introduces a trade-off between learning from data, but not to overfit to it. Thus, the confidence term regularizes the Pro-PINN model to reduce generalization error to unseen data, which could be noisy or irrelevant to the dynamics.

In a full-probabilistic setting, by expressing the normal distributions $\widehat{T}$ (temperature) and $\widehat{q}$ (heat) in terms of a standard normal distribution $Z \sim {{{\mathcal{N}}}}(0,1)$, one can show

14 $$\widehat{T}(x,y,t) := 	{T}_{conf}(x,y,t)Z+{T}_{pred}(x,y,t)\\ \widehat{q}(x,y,t) := 	{q}_{conf}(x,y,t)Z+{q}_{pred}(x,y,t).$$

Plugging the expressions of $\widehat{T}$ and $\widehat{q}$ of Eq. (14) in the PDE residual *L*_*PDE*_ in Eq. (13) we get

15 $${L}_{PDE-pred} := 	\frac{\partial }{\partial t}{T}_{pred}(x,y,t)\\ 	 + D\frac{{\partial }^{2}}{\partial {x}^{2}}{T}_{pred}(x,y,t)-{q}_{pred}(x,y,t),$$

with *L*_*PDE*−*conf*_ defined similarly and *L*_*total*_ being the weighted average of *L*_*prob*_, *L*_*PDE*−*pred*_ and *L*_*PDE*−*conf*_.

##### Active-sampling and robustness with data sparsity

Active learning (AL) is an iterative supervised ML method that queries a source of information interactively to label new data points for desired outcomes^40^. While we typically assume data availability through high spatial density camera images, this may not always hold true. To enable PINN models to handle occasional data sparsity reasonably well and to maintain the performance level, we introduce an active learning approach that iteratively selects device wall positions where PINN approximation performs the worst. These points guide optimal sensor (e.g., thermocouple) placement when camera data collection becomes impractical or impossible.

In our active sampling method, we select *m*_*AC*_ locations maximizing the objective function per iteration, where we use Mean Squared Error (MSE) (17) for temperature approximation. To prevent selecting samples too close to each other, we exclude locations within *d*_*p*_ of already sampled points and *d*_*b*_ of boundaries. Inspired by simulated annealing^41^, we optimize *d*_*p*_ by training a set of PINNs with different *d*_*p*_ values at each iteration, starting from identical initial weights and biases but using slightly different datasets influenced by the corresponding *d*_*p*_. The Results and discussion section demonstrates that active sampling achieves better training accuracy compared to a baseline of randomly selecting points.

In a real-life setting (e.g., in a factory or in the field), the measurement data used as input for the Pro-PINN model can be sparse, such as point-wise temperature captured by thermocouples. The uncertainty in both the data and the model’s prediction can be affected by this data sparsity. In this section, we discuss a method for decoupling Pro-PINN’s predictive uncertainty into data and model uncertainties, enabling quantitative analysis of model robustness against these uncertainties.

The uncertainty (Gaussian variance) ${\sigma }_{pred}^{2}$ in the variables (temperature and local heat loss) predicted by Pro-PINN can be divided into two components, *aleatoric* and *epistemic*, as described below^42^:

16 $${\sigma }_{pred}^{2}(x)={{\mathbb{E}}}_{i}[{\sigma }_{i}^{2}(x)]+Va{r}_{i}[{\mu }_{i}(x)],$$

where the first term on the right hand side of (16) denotes the *aleatoric* uncertainty that arise mainly due to uncertainty in the data (e.g., due to data procurement process), and the second term is the *epistemic* uncertainty which captures the uncertainty in the predictive model. We study the impact of data sparsity on both types of uncertainty. Here, ${{\mathbb{E}}}_{i}$ and *Var*_*i*_ denote the expectation and the variance, respectively, over samples *i*. The sample mean and variance are denoted by *μ*_*i*_ and ${\sigma }_{i}^{2}$.

##### Reconstruction-based validation

Validation of models solving inverse problems is difficult, primarily due to the lack of ground truth data for the cause being solved. However, since evaluating the accuracy of trained PINN models is crucial, we propose an indirect validation method. In this approach, measured temperature data serve as the input to the trained PINN model to estimate local heat loss. This estimated loss then becomes input to a multi-physics simulator platform (e.g., COMSOL, ANSYS), along with initial and boundary conditions, which reconstructs temperature from the input heat losses.

Define *e*_*T*_(*x*, *t*) ≔ ∥*T*_*gt*_(**x**, *t*) − *T*_*recon*_(**x**, *t*)∥^2^. Then, the prediction accuracy of the trained PIML model is evaluated using validation MSE and Largest Squared Error (LSE) defined as follows (adopted from Eqs. (3), (4)^27^, reproduced with permission from CIGRE):

17 $$MSE({T}_{gt},{T}_{recon}):=\frac{1}{| {\Omega }_{{{{\bf{x}}}}}| | {\Omega }_{t}| }{\sum}_{{{{\bf{x}}}}\in {\Omega }_{{{{\bf{x}}}}},t\in {\Omega }_{t}}{e}_{T}(x,t)$$

and

18 $$LSE({T}_{gt},{T}_{recon}):=\frac{1}{| {\Omega }_{t}| }{\max }_{{{{\bf{x}}}}\in {\Omega }_{{{{\bf{x}}}}}}{\sum}_{t\in {\Omega }_{t}}{e}_{T}(x,t),$$

where *T*_*gt*_ and *T*_*recon*_ denote the ground truth and reconstructed temperatures, respectively. Since the temperature distribution is the effect of the local heat losses, and the heat diffusion is a continuous process, sufficient accuracy (low error) in temperature prediction implies sufficiently accurate loss localization (i.e., distribution of heat) on the tank wall.

##### Comparison with classical and neural network-based solution approaches

Inverse problems are essentially optimization problems that can be solved using classical closed-form or iterative methods. In a deterministic setting, classical approaches employ a regularization method to diminish the effect of ill-posedness (e.g., non-existence, non-uniqueness or instability of solution). Most prominent regularization methods include the least-square method (e.g., Ridge regression or Tikhonov regularization), singular value decomposition method or variational approaches (e.g., total-variation regularization). In a probabilistic setting, the inverse problem can be viewed as learning the conditional probability of the cause given the noisy observations. The Bayesian approach is applied to use an assumed prior to learn this posterior distribution^43^. While the classical methods can provide closed-form solutions, there are some challenges that make these classical methods costly to implement. These challenges include the complexity of the nonlinear optimization problem, especially in a non-convex space, the convergence speed, and singularity (ill-posedness) in the inverse solutions. In the Bayesian approach, the main challenge is that it is computationally very expensive (an NP-hard problem to find an exact solution). While methods such as Markov Chain Monte Carlo (MCMC) can be employed to find approximate solutions^44^, they are sample-inefficient due to the need to multiple roll-outs of the forward model. The computation and time cost of these methods make them unsuitable for real-time applications.

A data-driven approach to solve inverse problems can be a good alternative to the classical methods due to their ability to give good sub-optimal solutions with a higher speed of convergence. In recent years, DNNs have been used to predict the forward problem, which is then used to iteratively predict the system parameters^12^. This approach solves the inverse problem in a deterministic setting with a shared network. We extend this setup to two variants of network architecture (separate and shared) and analyze their performances in the deterministic setting, and add the probabilistic framework and robustness analysis to the solution approach. Our probabilistic setting is computationally efficient, which predicts two Gaussian parameters rather than employing an expensive Bayesian inference method such as MCMC, as in ref. ^44^. Regularized ensemble Kalman inversion (EKI) method^45^ proposes to deal with dynamics where physics models typically do not have readily available adjoint capabilities.^46^ (DEKI) addresses the scalability of EKI to higher dimensions by introducing dropouts. Conformal inverse optimization approach^47^ proposes learning an uncertainty set for unknown parameters and prescribes new decisions that are well-aligned with human intuition.

We compare some of the aforementioned approaches with our end-to-end (E2E) approach, both qualitatively and quantitatively. For qualitative comparison, we ask if the approaches address the inverse problem in deterministic and probabilistic settings, if the approach can support higher-order polynomial dynamics, if full PDE solver is required to compute the solution and whether the method tracks full posterior (for probabilistic approaches). For quantitative comparison, we compare the validation MSE and time cost for training and inference of select approaches with ours.

## Results and discussion

In this section, we describe the experimental results that empirically establish the merits of the proposed end-to-end (E2E) approach for robustly solving inverse problems using the PIML method. To that end, we focus on a case study of finding the solution to the thermal diffusion problem by predicting the local heat loss distribution in electrical induction machines from thermal images measured from a heat-run test in a factory setup, as shown in Fig. 2b^27^. This case study represents a more industrially relevant application where ground truth validation is challenging and robust uncertainty quantification becomes essential for practical deployment. Table 1 shows the quantitative and qualitative results for this case study. Additionally, we provide some introductory benchmarking examples in the supplementary document to establish the foundation for the subsequent analysis of the thermal modeling problem of electrical induction devices in this paper. To that end, we establish benchmarks for UQ analyses on two nonlinear dynamics, which is described in the Benchmarking of UQ in Nonlinear Dynamics with Burgers Equation and Benchmarking of UQ in 2-D Nonlinear Dynamics with Compressible Navier Stokes Equations sections in the supplementary document. The results are shown in the “Comparison with Classical Methods” section.

**Table 1:** Quantitative and qualitative results for the case study of predicting local heat distribution in electrical induction devices

(a) Comparison of validation results for deterministic and probabilistic approaches.			
Device	Training setting	Validation MSE	Validation LSE
Power transformer	Deterministic	4.6 × 10^−2^	5.1 × 10^−1^
	Semi-Probabilistic	1.5 × 10^−2^	3 × 10^−1^
	Dropout (0.1)	7.9 × 10^−2^	8.7 × 10^−1^
	Dropout (0.5)	1.7 × 10^−1^	3.2
Shunt reactor	Deterministic	5.2 × 10^−2^	6.1 × 10^−1^
	Full-Probabilistic	1.4 × 10^−2^	3 × 10^−1^

(b) Qualitative comparisons of inverse problem methodologies.					
Approach	Deterministic	Probabilistic	Polynomial extension	Full PDE solver required	Tracks full posterior
Tikhonov	Yes	No	No	No	-
Bayesian MCMC	No	Yes	No	Yes	Yes
BNN-MC	No	Yes	Yes	No	Yes
BNN-Dropout	No	Yes	Yes	No	No
EKI	Yes	No	No	Yes	-
**E2E (ours)**	Yes	Yes	Yes	No	No

(c) Performance and training time-cost benchmarking on power transformer data.		
Approach	Validation MSE	Training time (min)
Tikhonov	6.0 × 10^−2^	4
Bayesian MCMC	2.9 × 10^−2^	850
BNN-MC	0.9 × 10^−2^	189
BNN-Dropout	6.8 × 10^−2^	38
EKI	4.9 × 10^−2^	45
**E2E (ours, deterministic)**	4.6 × 10^−2^	18
**E2E (ours, probabilistic)**	1.5 × 10^−2^	25

(d) Inference time-cost benchmarking on power transformer data.	
Approach	Inference time (ms) (per sample)
Tikhonov	5
BNN-MC	2975
BNN-Dropout	416
EKI	8
**E2E (ours, deterministic)**	8
**E2E (ours, probabilistic)**	9

### Results for thermal diffusion problem in electrical induction machines: training and validation of deterministic PINN

**Fig. 5:**
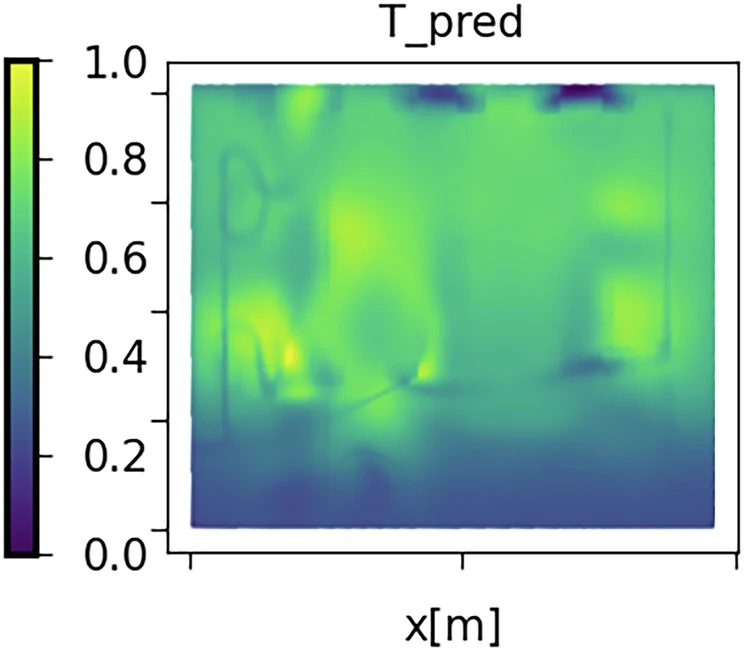
The trained deterministic PINN model is able to predict the continuity of the temperature distribution. In this figure, T_pred_ is the predicted temperature distribution on the device’s wall. We observe that the pixels of irrelevant data such as wires on the device wall are suppressed in the prediction and also the underlying continuity in temperature distribution is captured despite the irrelevant data measured from some regions of the wall, as shown by the blue columns in Fig. 3a.

Figure 5 shows the temperature distribution reconstructed using deterministic PINN with myopic training. Here *T*_*pred*_ is the temperature reconstructed from the heat distribution predicted by PINN, using as inputs the measured temperatures on the device wall, as shown in Fig. 3a. We observe from Fig. 5 that the model successfully learns the continuity of the underlying dynamics satisfactorily, even in the presence of unreliable or noisy measurements, e.g., of some artifacts on the device wall, such as partially visible cables in Fig. 5, or of some regions on the wall not reflecting the temperature distribution due to heat diffusion process reliably, as is depicted by the blue columns in Fig. 3a. While it partially suppresses undesirable data features (noise or pixels not participating in heat diffusion, like images of cables), it doesn’t remove them completely, indicating some *overfitting*.

**Fig. 6:**
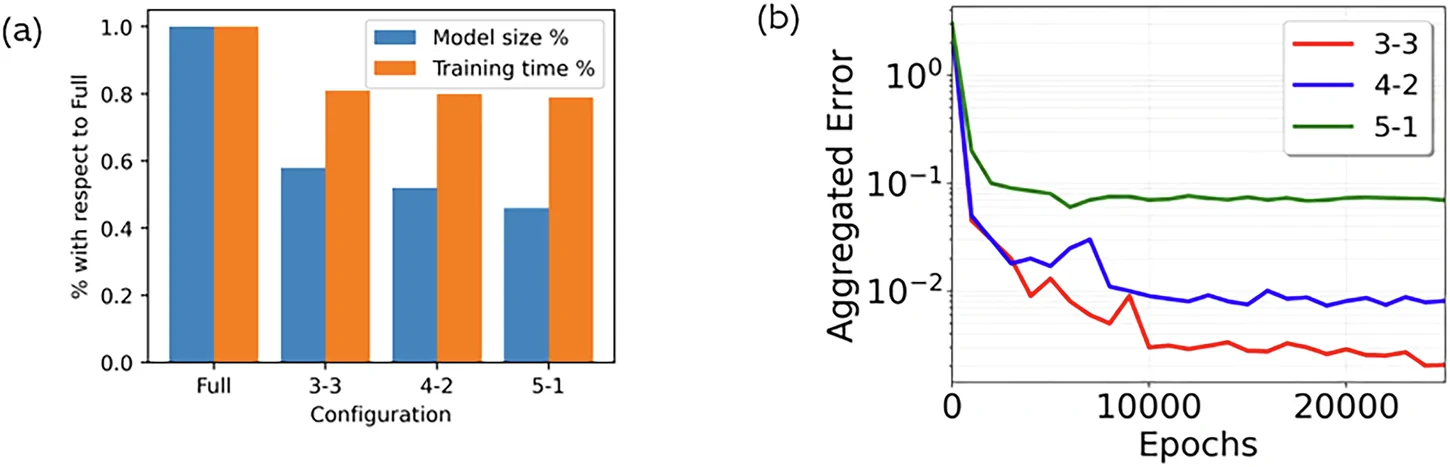
Effect of sharing of layers in deterministic PINN on model sizes (in the form # shared layers – # separate layers), training time, and training errors (adapted from Fig. 7^27^, reproduced with permission from CIGRE). **a** Model size / Training time. **b** Training errors.

Figure 6 (adapted from ref. ^27^) presents a comparative study of layer sharing in deterministic PINN. As expected, results show inherent trade-offs: while model size and training time decrease with more shared layers, training error increases.

**Fig. 7:**
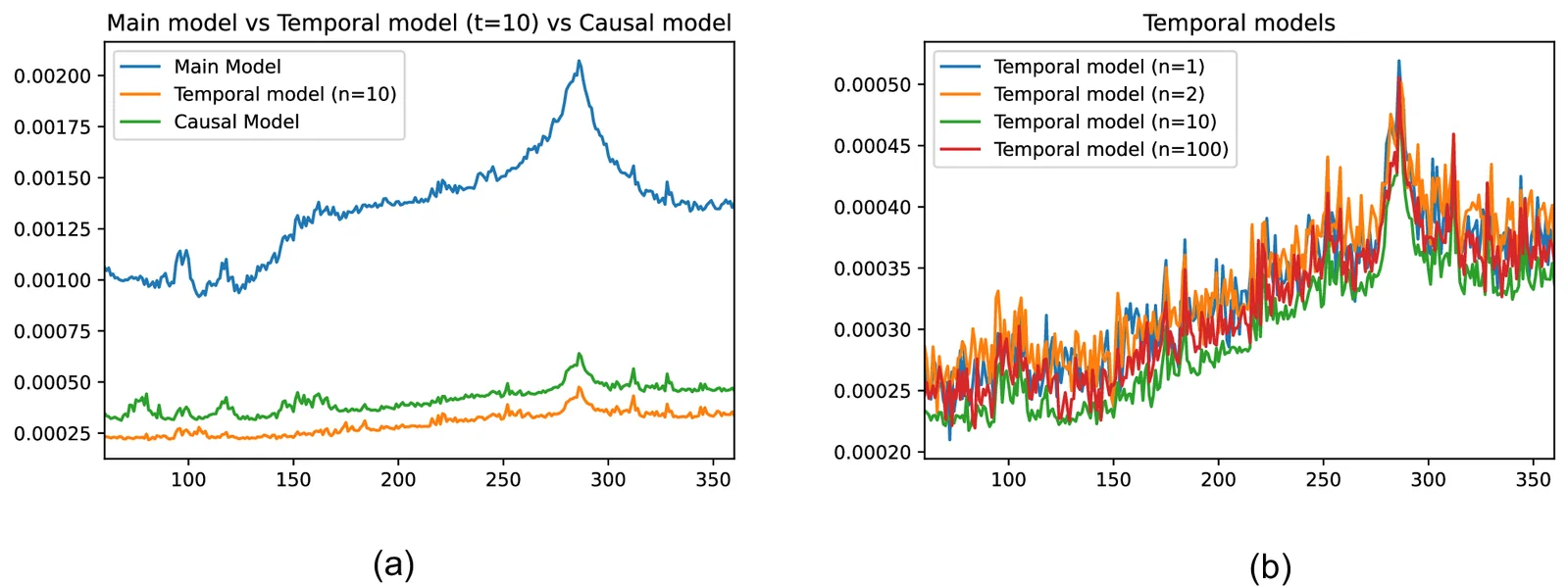
Temporal gradient-based history-aware training of deterministic PINN. **a** Training losses with and without history-aware training. **b** History-aware training losses with different window sizes *n*.

Figure 7 compares temporal-gradient history-aware training (*Temporal model*) against myopic (*Main model*) and *causal* training^39^ (*Causal model*). Temporal-gradient approach with *n* = 10 outperforms baselines and is computationally cheaper than causal training by using only recent past information. Figure 7b demonstrates that larger windows (size 100) increase training error compared to size 10, suggesting that irrelevant distant past information may harm the training process.

### Results for thermal diffusion problem in electrical induction machines: training and validation of Pro-PINN

**Fig. 8:**
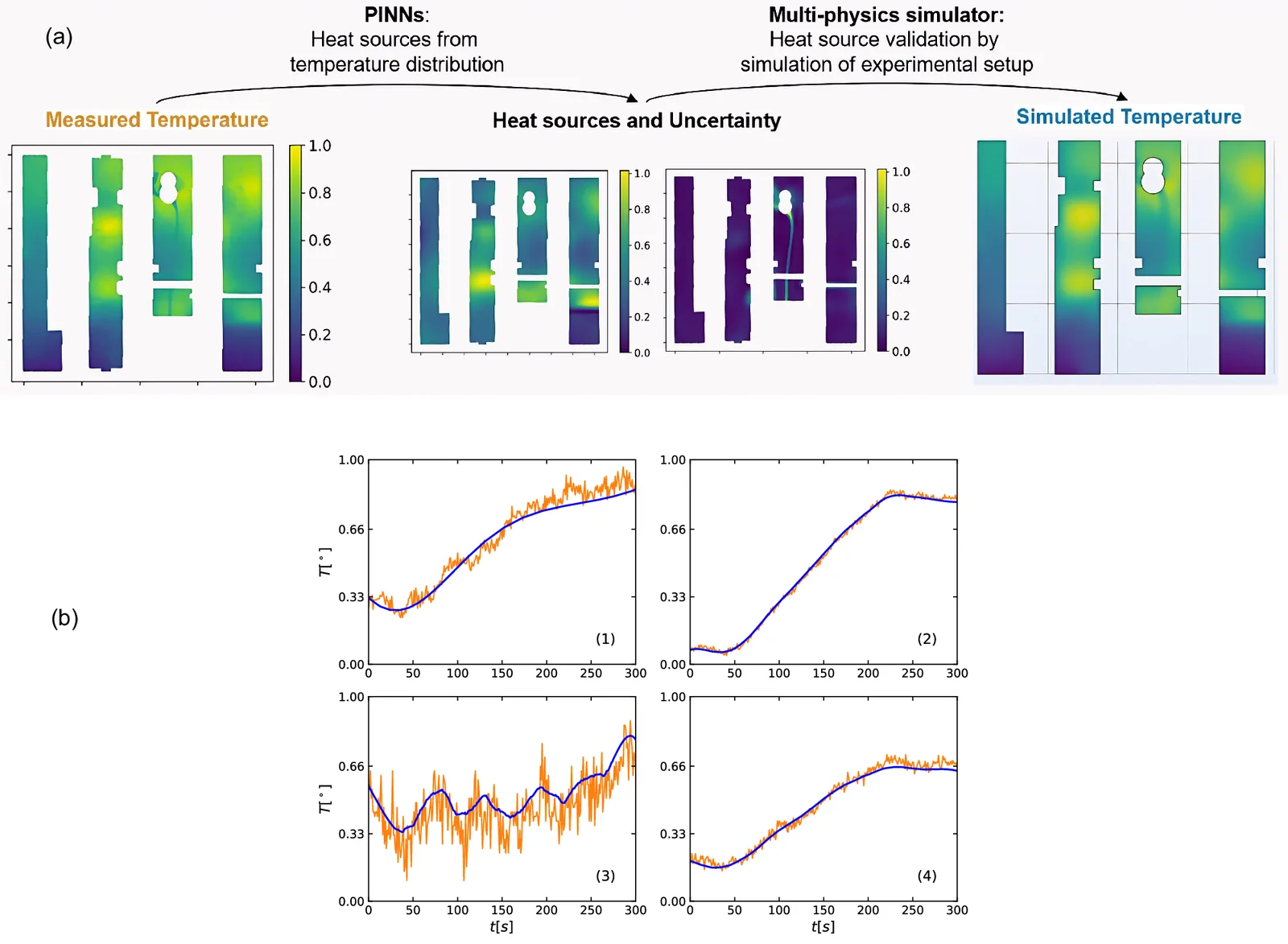
Validation of trained Pro-PINN models to estimate local heat distribution in shunt reactors by reconstructing the temperature distribution and spatiotemporal comparison with measured temperatures. **a** Reconstruction-based validation process. The images are captured at *t* = 300*s* to reflect the spatial distributions. **b** Comparison of measured (orange curves) and reconstructed (blue curves) temperatures over time. The points (1) [0.35, 0.65], (2) [0.35, 0.3], (3) [0.6, 0.65] and (4) [0.85, 0.65] are shown in Fig. 8a.

Pro-PINN provides quantification of the confidence in predicted heat loss distribution, enhancing interpretability by directing human experts to regions needing further investigation. Figure 8 shows the reconstruction-based validation of full Pro-PINNs for shunt reactors at a given time instant as well as over a time range. Figure 8a shows the prediction of heat localization and its uncertainty by Pro-PINN at a given time instant using thermal distributions as inputs, followed by reconstruction of the temperature distribution using a multi-physics simulator using the predicted heat distribution as the inputs. The accuracy of the prediction of Pro-PINN over time is demonstrated in Fig. 8b where the measured temperature (orange curves) and the temperatures reconstructed (blue curves) from the heat predictions by Pro-PINN are compared at specific spatial coordinates on the device wall. Since the underlying dynamics is spatio-temporally continuous, this establishes the satisfactory performance of Pro-PINN in predicting the heat that causes a denoised version of the measured temperature.

Table 1a a presents the validation MSE and validation LSE for the power transformer and shunt reactor, respectively. The results demonstrate that incorporating uncertainty quantification through probabilistic methods (semi-Pro-PINN for transformer and full Pro-PINN for reactor) leads to notably better prediction accuracy compared to the deterministic approach (e.g., approximately 73% reduction in MSE for shunt reactor) for both devices. It also reveals that insertion of dropouts in the deterministic setting worsens the performance, and the performance deteriorates with a higher rate of dropout (the performance with a dropout rate of 0.5 is worse than that with a dropout rate of 0.1). Additionally, we provide qualitative results in the Additional Results section in the supplementary document, where Fig. 8 shows a low dropout rate of 0.1 is insufficient to prevent overfitting and remove artifacts from the prediction. Conversely, a high rate of 0.5, while effective in removing these artifacts, leads to an oversmoothed prediction that loses important physical details, demonstrating the challenge of manually tuning such regularization. This suggests that an uncertainty framework around the dynamics is more suited to reduce overfitting to irrelevant data that do not come from the dynamics.

### Results for active sampling and robustness with data sparsity

Model uncertainty comprises data uncertainty (aleatoric) and model’s internal uncertainty (epistemic). We observe through experiments that while prediction confidence decreases with sparser data due to increased data uncertainty, the model’s internal uncertainty remains relatively stable. This enables robust predictions with reduced data, where we use 0.13% (160 samples) of original samples to get a validation error of 1.5 × 10^−3^, which is an increase by 15%.

An alternative strategy involves replacing thermal camera inputs with limited point-wise temperature readings from the transformer tank-wall. Since both sensor quantity and arrangement affect results, we develop a strategic placement method using the uncertainty metric *q*_*conf*_ defined in Eq. (14). This metric is advantageous, as it can be computed directly by the neural network without requiring prior temperature values, providing a sensitivity metric that correlates uncertainties with specific wall locations.

**Fig. 9:**
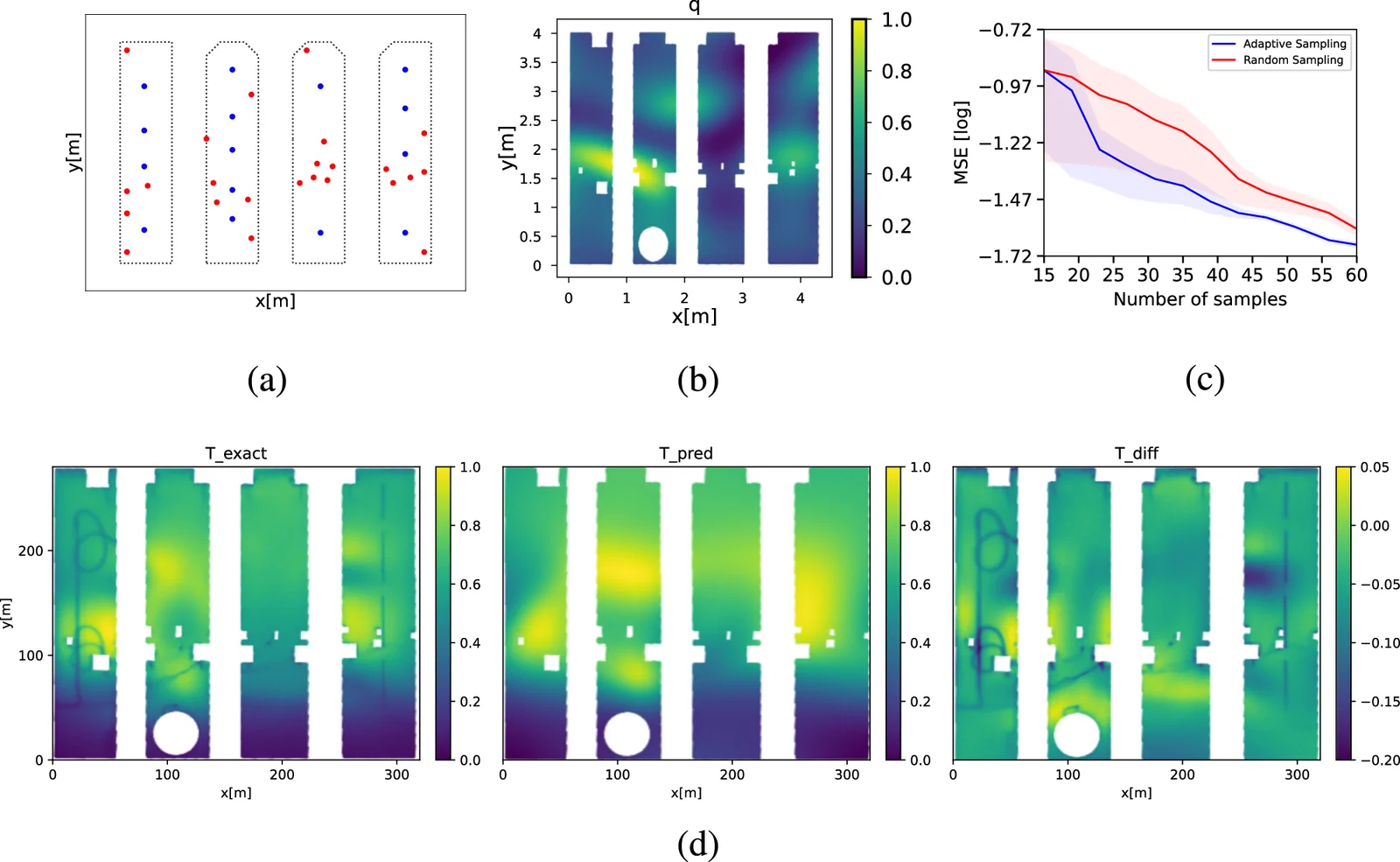
Results for the active sampling method. **a** Samples found by the active sampling method. **b** Prediction of local heat distribution using an active sampling method. **c** Comparison of MSE between adaptive and random sampling strategies. **d** Measured and predicted temperature distribution using the active sampling method, and their difference.

The rationale for selecting confidence as the metric lies in its ability to be computed directly by the neural network without any prior knowledge of the actual temperature values. This approach offers a sensitivity metric that correlates uncertainties in temperature variations with specific locations on the tank wall. The methodology begins with placing a limited set of fixed sensors (e.g., 15) on the tank wall, followed by an iterative process for additional sensors. In this procedure we do the following steps: (i) configure initial sensor placements (denoted by the blue points in Fig. 9a), (ii) train PINN following procedure detailed in Training and validation of PINN models section, (iii) assess the model’s confidence in its predictions across *N* points to identify *m* lowest-confidence points based on uncertainties, and (iv) add these *m* points to training dataset for further retraining. We achieve satisfactory performance while avoiding too much retraining by using *N* = 100, 000 uniformly distributed points and *m* = 4 sensors per iteration (minimum 0.04*m* spacing). The final sensor distribution after 6 iterations is shown in Fig. 9a (red points). From Fig. 9b and d, we observe successful identification of temperature hot spots (*T*) and local heat loss (*q*) despite limited observation points.

To validate our adaptive sampling approach, we conduct a comparative analysis against random sampling across different measurement points. Figure 9c shows the MSE of temperature prediction versus sample numbers for both strategies, with shaded regions representing one standard deviation from 5 independent trials. Results demonstrate adaptive sampling’s ability to achieve lower MSE and reduced variance across 15−55 points. The approach shows particularly rapid error reduction in early stages (15−25 samples), indicating efficient identification of informative measurement locations. The active-sampling-based approach demonstrates more consistent performance compared to the random sampling method used as the baseline, where the former achieves narrower confidence bands than the latter. The superior performance stems from the method’s ability to iteratively identify regions of high uncertainty, strategically placing sensors for maximum information gain. This targeted approach enables accurate temperature field reconstruction with 30% fewer measurement points than random sampling for equivalent accuracy.

### Comparison with classical methods

In this section, we compare our end-to-end (E2E) approach with a range of deterministic as well as probabilistic classical methods to benchmark the performance and cost of these methods for the power transformer test case. For the fairness of comparison, we report the training and inference times as measures of the computational cost rather than convergence rates, since the underlying optimization methods are quite different, which affects the convergence rates. For example, for one benchmarking method, one iteration requires a simple gradient propagation, another method requires solving a set of partial differential equations, and yet another approach requires solving a linear system. The qualitative as well as the quantitative results show that the E2E methods perform competitively with the baseline methods, often demonstrating better balance in performance and computation cost than the baseline methods.

A qualitative comparison for our end-to-end (E2E) framework with several baseline methods is presented in Table 1b. The comparison is based on key properties, including whether the methods provide deterministic or probabilistic solutions, allow approximations of high-order polynomial dynamics, require the use of a full solver, and keep track of posteriors for probabilistic approaches. The last two properties are relevant for computation costs, where the requirement of a full solver and need to keep track of the posterior distributions make a method costly. For instance, Table 1b shows that Tikhonov regularization is purely deterministic and does not require the use of a full solver, making it computationally inexpensive, while methods like Bayesian Neural Network Monte-Carlo (BNN-MC) offer probabilistic setting but keep track of the posterior, making the computational cost high. In contrast, the proposed E2E framework is the only approach that addresses all the evaluated criteria, offering a unified framework for the deterministic as well as probabilistic settings, balancing between good performance and relatively low computational cost due to no requirement of a full solver and keeping track of the posterior distribution, since it rather estimates the parameters of the Gaussian posterior. As described in the End-to-end automated and robust system discovery for electrical dynamical systems section, the E2E method is capable of handling uncertainty quantification for problems characterized by polynomial dynamics. For BNN-based methods, a probabilistic approximation of the solution to the inverse problem with polynomial dynamics is provided by construction.

Table 1c shows that our proposed E2E method performs competitively with all baseline methods, both in deterministic and probabilistic settings. In particular, it achieves better performance (i.e., lower validation error) than all baselines except BNN-MC. The merit of E2E probabilistic becomes more apparent when we compare the computation cost of the method with Bayesian baselines, which reveals that the BNN-MC is more computationally costly, requiring higher training as well as inference time (Table 1d) compared to E2E probabilistic method.

The benchmarking approaches in Table 1c represent a broad spectrum of techniques for solving inverse problems, each having a distinct trade-off between predictive accuracy (measured by validation MSE) and computational cost (measured by training time). We summarize the benchmarking methods and a comparative study below.

#### Deterministic baseline methods

We apply Tikhonov regularization for a deterministic approach, which is serves as a classical baseline for regularization. We implement the Tikhonov method by solving the augmented normal equations using conjugate gradient descent, as described in ref. ^48^. Its performance, however, is critically dependent on the manual selection of a regularization parameter, *γ*, as noted in ref. ^49^. For our problem, we notice that even a small value (0.01) has a significant impact on the accuracy, and oversmoothing of physical features. As our second deterministic baseline, we apply Ensemble Kalman Inversion (EKI)^50^, which presents a derivative-free, iterative optimization framework whose cost scales with the ensemble size (J = 100 in our case) and the number of iterations. The method iteratively updates the ensemble through a prediction-correction scheme, computing empirical covariance matrices at each iteration. Our implementation requires approximately 20 iterations to converge, taking 45 minutes of training time, following the reference methodology proposed in ref. ^50^. The method achieves a validation MSE of 4.9 × 10^−2^, offering a reasonable balance between accuracy and computational cost. A key characteristic of EKI is the *subspace property*, which constrains the solution to the linear span of the initial ensemble, making its performance highly dependent on the quality of the initial guess.

#### Probabilistic baseline methods

We use Bayesian MCMC, BNN-MC, and BNN-Dropout as the baselines for the probabilistic approach. It is clear from the results that the implicit regularization offered by the stochastic formulation of our approach is more natural and offers a distinct improvement in accuracy. Fully probabilistic methods like Bayesian MCMC^51^ aim to approximate the entire posterior distribution of the unknown heat source. We implement full Bayesian inference using the preconditioned Crank-Nicolson MCMC method with a Gaussian process prior for sampling the posterior distribution of heat sources, following^44^. While theoretically robust, MCMC is exceptionally costly, often requiring tens of thousands of sequential forward model evaluations after an initial *burn-in* period of discarded samples, rendering it impractical for real-time applications. The process requires approximately 50000 samples with a burn-in period of 10000 iterations to adequately explore the posterior distribution in our infinite-dimensional parameter space. Training time reaches 850 minutes on our hardware. This aligns with findings in ref. ^51^, which reports that traditional MCMC methods for PDE-constrained inverse problems can be several times slower due to the need for repeated forward model evaluations. The high computational cost makes MCMC less practical for real-time industrial applications, despite a higher rigor in uncertainty quantification. Recent studies on surrogate-accelerated MCMC have shown that replacing expensive PDE solvers with neural network surrogates can reduce costs, but at the expense of introducing approximation errors. Neural network-based Bayesian methods exhibit a similar problem: the traditional Monte Carlo (MC) approach places distributions over each network weight, doubling the number of trainable parameters and leading to high computational and memory costs. Following^52^, we implemented BNN-MC using Hamiltonian Monte Carlo (HMC) for posterior estimation over network weights. Our implementation employed 8 hidden layers with 128 neurons per layer, using Gaussian priors with zero mean and unit variance on all weights and biases. HMC sampling has been performed with 100 leapfrog steps per iteration and step size *ϵ* = 0.001. We collect 10000 posterior samples after a burn-in of 2000 iterations, requiring 189 minutes of training time. In contrast, the MC Dropout(BNN Dropout) approximation offers a cheaper alternative by keeping dropout active during inference, as suggested in ref. ^53^, but this efficiency comes at the cost of theoretical rigor. During training, a dropout rate *p* = 0.1 has been applied to all hidden layers for 50,000 epochs, requiring 38 minutes. At inference time, dropout remains active, and we perform 100 forward passes to approximate the posterior predictive distribution. This yields an inference time of approximately 400 ms per sample, faster than full BNN-MC but still substantially slower than deterministic methods.^54^ show that while dropout helps prevent overfitting, it provides less theoretically grounded uncertainty estimates compared to full Bayesian treatments.

#### Comparison of benchmarking methods

The quantitative results in Table 1c align with the computational considerations discussed above, showing distinct performances. The most computationally expensive methods, BNN-MC and Bayesian MCMC, deliver the highest accuracy, achieving validation Mean Squared Errors (MSEs) of 0.9 × 10^−2^ and 2.9 × 10^−2^, respectively. BNN-MC achieves the best validation among all approaches, but at a significant computation cost. Inference requires propagating input through the network multiple times with different weight samples to estimate the posterior predictive distribution, resulting in 3000 ms per sample. Conversely, Tikhonov regularization and the BNN (Dropout) approximation are the least accurate, with MSEs of 6.0 × 10^−2^ and 6.8 × 10^−2^. This suggests that for a complex problem such as heat source identification in a shunt reactor, their underlying simplifying assumptions are overly restrictive. The Ensemble Kalman Inversion (EKI) provides a moderate balance, with an MSE of 4.9 × 10^−2^. In this context, our proposed E2E probabilistic framework demonstrates a highly competitive profile. It achieves a validation MSE of 1.4 × 10^−2^, approaching the accuracy of the best-performing yet computationally prohibitive BNN-MC method. The computational costs are measured in terms of training times in Table 1c as well as mentioned in the description of the baseline methods given above, where it shows that our E2E approaches outperform all baselines except Tikhonov, which achieves the lowest training cost.

To illustrate the merit of our proposed E2E deterministic as well as probabilistic methods in balancing the performance and computational cost, in addition to the training times given in Table 1c, we compare in Table 1d the inference time costs for the classical deterministic approaches, Tikhonov and EKI, with our deterministic E2E approach, and two Bayesian methods, the best-performing BNN-MC and computationally cheaper BNN-Dropout with our probabilistic E2E approach. We observe that E2E approach largely outperforms probabilistic baselines and performs competitively with deterministic baselines. In the comparison, we take into account the inference times for the shunt reactor example described in the Results for Thermal Diffusion Problem in Electrical Induction Machines: Training and Validation of Pro-PINN section. While the E2E approach requires a single evaluation for the parameters (mean and variance) of the learned parametric distribution (Gaussian), BNN-based approaches require sampling from learned distribution of weights and biases to obtain uncertainty estimates as statistics, leading to an increase in the computational cost (time). We infer from Tables 1c and d that our E2E approach is more efficient in balancing the performance with the cost compared to the baselines.

### Discussion

We establish through theoretical analysis and empirical evidence five key merits of PIML in heat loss localization: (i) the importance of data processing in prediction accuracy, (ii) an efficient way to balance trade-offs among prediction accuracy, computation and storage costs through PIML model architecture variants, (iii) a history-aware training method improving accuracy by incorporating relevant past information, (iv) a computationally efficient UQ method enhancing model interpretability and enabling robust sampling analysis, and (v) an indirect, reconstruction-based validation using multi-physics simulation. This methodology shows potential for extension to components where measurement data procurement is challenging or of lower quality.

In the context of estimating heat losses from temperature measurements using inverse problems, it is crucial to recognize that achieving a perfect fit between model predictions and experimental data may not be desirable. A perfect fit could imply that the model is capturing not only the underlying physical phenomena but also the noise inherent in the measurements. This phenomenon, often referred to as overfitting, can lead to misleading conclusions about the system’s behavior. In practical applications, especially in thermal imaging and heat transfer analysis, measurement noise is an unavoidable reality that can influence the accuracy of parameter estimations. The presence of noise necessitates a careful balance in model training, where the goal should be to capture the essential thermal dynamics while remaining robust against measurement uncertainties. If the model aligns too closely with noisy data, it risks losing its predictive capability and generalizability. Instead, a more prudent approach involves leveraging regularization techniques or incorporating prior knowledge about the system to mitigate the effects of noise. This ensures that the model remains focused on the underlying physics rather than fitting random fluctuations in the data. It has been shown that thermal measurement noise could pose a challenge in estimating boundary conditions and heat loss rates^55^. In our approach, by leveraging PIML and by introducing a probabilistic framework, we manage to capture the dynamical behavior of the heat losses with good accuracy and robustness. The predictive accuracy of the probabilistic approach (Pro-PINN) is shown and compared with that of its deterministic counterpart in Table 1a. However, instead of reducing the accuracy further, which may lead to overfitting, this approach maintains the robustness of prediction by leveraging an inherent trade-off in its training method, which is reflected in Fig. 8, which depicts the de-noising effect in the temperatures reconstructed from predicted heat loss without overfitting to the noise present in the measured temperature, which requires no additional steps of filtering of the training data.

In inverse problems with noisy measurements, achieving a perfect model-data fit is not desirable as it indicates overfitting, i.e., capturing not just underlying physics but also measurement noise, which can lead to misleading conclusions about system behavior. When working with noisy measurement data, it is crucial to capture essential thermal dynamics while maintaining robustness against uncertainties to ensure generalizability. Previous research has shown thermal measurement noise impacts boundary condition and heat loss rate estimation^55^. Our probabilistic PIML approach (Pro-PINN) successfully captures heat loss dynamics with both accuracy and robustness. Table 1a and Fig. 8 establish that Pro-PINN achieves reduced overfitting and more accurate and robust prediction in the presence of noisy data.

## Conclusion

This paper presents an end-to-end approach to predict the cause of the observed effect in electrical and power dynamical systems and quantify the uncertainties for a class of dynamics (governed by polynomial spatiotemporal ODEs or PDEs). To that end, we use Physics-Informed Machine Learning (PIML) approach with Neural Networks as the predictive models, develop the theory for the uncertainty, and address the robustness of the predictions in the presence of sparse data. Our empirical results with simulated as well as real data demonstrate PIML’s ability to accurately predict heat distribution beyond current physics simulation capabilities, while its uncertainty quantification enhances prediction accuracy and interpretability, enabling expert investigation of root causes. It introduces an end-to-end approach to balance the cost and performance, hence providing solutions that can be applied to near-real-time applications. While the history-aware training of models makes the training data-efficient, the quantitative analysis of the robustness and uncertainties makes the solutions more accurate and interpretable by users. By providing a unified framework for automated and robust system discovery in both deterministic and probabilistic settings, our work establishes a foundation for future research combining domain knowledge with data-driven methods for complex electrical assets, and thus lays the ground for more enhanced manufacturing and asset monitoring processes using combined data and domain-knowledge-driven approaches. The end-to-end approach to automated and robust system discovery introduced in this paper can contribute to future research in modeling multi-physics systems, such as the modeling of current-carrying capacity of underground and overhead power cable lines that require modeling of the physics involving the electrical, electromagnetic, and thermal dynamics, and modeling electrical contacts in electrical and electronic devices requiring modeling of pressure, electrical, and thermal dynamics.

## Supplementary information

Transparent Peer Review file

Supplementary Information

## Data Availability

The data used in case study are not publicly available due to potential compromise of the data privacy. However, some of the benchmarking experiments mentioned in the paper use simulated data, for which we have provided standalone codes mentioned in Code Availability Statement section.

## Code availability

The source codes for the experiments mentioned in the main manuscript as well as in the supplementary document that did not use any confidential data have been shared in ref. ^56^. A README file for each set of experiments describing the data, the models, and the dependencies for running the codes has also been provided.
