# Supplementary material for: End-to-end robust system discovery in electrical dynamical systems using scientific machine learning
Source: Commun Eng. 2026 Jun 16;5:172. doi: 10.1038/s44172-026-00689-2 (PMC13645738; doi:10.1038/s44172-026-00689-2)
Supplement: Supplementary file 1 [file 44172_2026_689_MOESM1_ESM.pdf]

nature portfolio

Peer Review File

End-to-end robust system discovery in electrical dynamical systems using scientific machine learning

Corresponding Author: Dr Jhelum Chakravorty

This file contains all reviewer reports in order by version, followed by all author rebuttals in order by version.

Attachments originally included by the reviewers as part of their assessment can be found at the end of this file.

Version 0:

Reviewer comments:

Reviewer #1

(Remarks to the Author)
The paper proposes an original approach for inverse problems in dynamical electrical systems, introducing probabilistic PINNs in this domain, which appears to be a novel contribution. The manuscript includes theoretical development, model architecture, and a detailed explanation of the uncertainty quantification process. The work is directly applicable to condition monitoring in electrical machines, transformer heat loss estimation, and other industrial systems where data may be sparse or noisy. The paper is generally well-organized.
However, there are some points to be considered:
1. There is little to no comparison with alternative state-of-the-art methods outside of PINNs. A quantitative comparison with more traditional methods would strengthen the proposed methodology.
2. The models are demonstrated for 2D spatial problems and relatively short time spans. The scalability of the approach to 3D systems or longer-term monitoring tasks in industrial settings remains unclear.
3. The validation relies heavily on the assumption that the multi-physics simulators are accurate representations of the real-world systems, which may not always hold. A discussion of the limitations and possible biases introduced by this indirect validation is missing.
4. Providing more details about the noise characteristics, calibration, and accuracy of the thermal imaging used in experiments would improve transparency.
5. While mathematically thorough, some derivations (e.g., in Section II-B) may be hard for readers without a strong background in differential equations and stochastic processes. Additional explanations are needed.

Reviewer #3

(Remarks to the Author)
Please see attachment.

Reviewer #4

(Remarks to the Author)
I co-reviewed this manuscript with one of the reviewers who provided the listed reports. This is part of the Communications Engineering initiative to facilitate training in peer review and to provide appropriate recognition for Early Career Researchers who co-review manuscripts.

Version 2:

Reviewer comments:

Reviewer #1

(Remarks to the Author)
- The abstract should highlight specific quantitative outcomes (accuracy improvements, computational gains) rather than

remaining general.
- While several classical and neural approaches are mentioned, the quantitative comparison section could be expanded. Including explicit numerical performance tables (e.g., computation time, rates) would strengthen the validation.
- The discussion of practical usage should be more detailed.
- The mathematical derivations could be shortened or moved to an appendix to improve readability.
- Include a table with quantitative benchmarking results. Metrics such as training/inference time, validation, and model convergence speed compared to baseline methods would support claims of efficiency.
- The conclusion section could better emphasize the novelty and future potential.
- Minor grammatical and stylistic edits must be considered.
- Readability must be improved.

Reviewer #3

(Remarks to the Author)
The reviewer thanks the authors for their responses. The replies are comprehensive and satisfactorily address the questions. The reviewer recommends this paper to be accepted.

Reviewer #4

(Remarks to the Author)
I co-reviewed this manuscript with one of the reviewers who provided the listed reports. This is part of the Communications Engineering initiative to facilitate training in peer review and to provide appropriate recognition for Early Career Researchers who co-review manuscripts.

Version 3:

Reviewer comments:

Reviewer #1

(Remarks to the Author)
Thank you for considering my suggestions. I have no further comments.

Open Access This Peer Review File is licensed under a Creative Commons Attribution 4.0 International License, which permits use, sharing, adaptation, distribution and reproduction in any medium or format, as long as you give appropriate credit to the original author(s) and the source, provide a link to the Creative Commons license, and indicate if changes were made.
In cases where reviewers are anonymous, credit should be given to 'Anonymous Referee' and the source.
The images or other third party material in this Peer Review File are included in the article's Creative Commons license, unless indicated otherwise in a credit line to the material. If material is not included in the article's Creative Commons license and your intended use is not permitted by statutory regulation or exceeds the permitted use, you will need to obtain

permission directly from the copyright holder.
To view a copy of this license, visit https://creativecommons.org/licenses/by/4.0/

Authors' response to reviewers' comments

The authors thank the reviewers for their comments. In this document we address the reviewers' comments in detail.

1 Comment 1

Comment: *The paper acknowledges the importance of avoiding overfitting in inverse problem settings.*

It would be beneficial if the authors could discuss or experiment with standard regularization techniques commonly used in neural networks, such as dropout and batch normalization,. It would be interesting to assess whether these methods lead to notable improvements in prediction robustness and generalization, particularly under data noise or sparsity.

Response: We thank the reviewer for the comment. We have run additional experiments with dropout introduced in the deterministic setting where we consider different values for the dropout rate (0.1 and 0.5), but they were either failing in removing the artifacts from the comparison or oversmoothing the approximation. This suggests that an uncertainty framework around the dynamics is more suited to reduce overfitting to irrelevant data that does not come from the dynamics. We have shown qualitative comparison results in Fig. 18. For ease of exposition, we have highlighted the text relevant to this comment in brown.

2 Comment 2

Comment: *In Figure 11(b), it appears that the neural network training has not yet converged. It is*

recommended that the authors extend the training for additional epochs to determine whether the loss curve eventually flattens. This could help confirm whether the observed trends are due to model capacity limitations or insufficient training duration.

Response: The authors thank the reviewer for pointing this out. We have updated Fig. 14(b) with a greater number of epochs to show the convergence. The updated figure reveals that the plot in the previous version of the manuscript had insufficient training duration, which is corrected in this plot that shows clear convergence now. This figure conveys the message that there is a tradeoff between performance and memory cost. Higher number of shared layers reduces the memory cost, but the performance gets inferior (giving higher error). For ease of exposition, we have highlighted the text related to this comment in teal.

3 Comment 3

Comment: *The current numerical experiments do not include comparisons with other neural network-based approaches or classical inverse problem methods. Including such benchmarks would provide a more complete perspective on the advantages of the proposed framework. Additionally, given that neural networks are particularly well-suited to high-dimensional problems, it would strengthen the paper to include a higher-dimensional example to further illustrate the effectiveness and scalability of the proposed method.*

Response: We thank the reviewer for the comment. To address this, we have included some classical and neural network-based approaches in Section V-F. In addition, we did more experiments with some of these methods as baselines, and added qualitative and quantitative comparisons in VI-F.

Regarding the comment on higher dimensionality of the problem, we would like to emphasize that the goal of this paper is to address the end-to-end robust solutions for inverse problems in both deterministic as well as probabilistic settings, with an efficient balance between the performance and the compute (time) cost, and to provide a unified framework to do so for a class of polynomial dynamics. The paper does not focus on tackling very high-dimensional dynamics, primarily because such systems are not very common in electrical and power systems, which is the scope of this paper. Also, it is noteworthy that although neural networks are indeed efficient in learning nonlinear functions in high-dimensional parameter space, the main challenge that this paper is trying to address is the complexity of finding the solutions in inverse problems involving dynamics. In this context, it is more relevant to address the dimensionality of dynamics and not the parameter space. Hence, to address the reviewer's comment on higher dimensional problems, we have included an additional simplified benchmarking on an inverse problem for 2-dimensional (2-D) compressible Navier Stokes dynamics (also included this in

the abstract of the paper). Similar equations are used to study the dynamical behavior of gas insulated circuit breakers. We have added the results in Section VI-B.

For ease of exposition, we have added all additional texts in response to this reviewer's comments in blue.

4 Additional remarks from the authors

The authors thank the reviewers for catching the typos. We have corrected the typos in the revised manuscript.

Additionally, for a better flow of the text, we made a few editorial changes in the paper. Also, we included a note on code availability in Section VI-H. We have highlighted them in magenta.

Authors' response to reviewers' comments

The authors thank the reviewers for their comments. In this document we address the reviewers' comments in detail.

Reviewer 1's comments

Comment 1: *There is little to no comparison with alternative state-of-the-art methods outside of PINNs. A quantitative comparison with more traditional methods would strengthen the proposed methodology.*

Response: We thank the reviewer for the comment. To address this, we have included some classical and neural network-based approaches in Section V-F. In addition, we did more experiments with some of these methods as baselines, and added qualitative and quantitative comparisons in VI-F. For ease of exposition, we have added all additional texts in response to this reviewer's comments in blue.

Comment 2: *The models are demonstrated for 2D spatial problems and relatively short time spans. The scalability of the approach to 3D systems or longer-term monitoring tasks in industrial settings remains unclear.*

Response: We thank the reviewer for the comment. To address this point, we have added a discussion in Section III-B. For ease of finding them, we have added the text in orange.

Comment 3: *The validation relies heavily on the assumption that the multi-physics simulators are accurate representations of the real-world systems, which may not always hold. A discussion of the limitations and possible biases introduced by this indirect validation is missing.*

Response: We thank the reviewer for this insightful comment. To address this, we have added a discussion on the limitations and potential biases of our indirect validation method, acknowledging the role of model discrepancy, in Section I-D. For ease of review, the new text has been highlighted in cyan.

Comment 4: *Providing more details about the noise characteristics, calibration, and accuracy of the thermal imaging used in experiments would improve transparency.*

Response: We thank the reviewer for the comment. In response, we have added a discussion in Section III-B on the accuracy and noise sensitivity of the thermal camera and calibration of thermal camera measurements with the readings from thermocouples and added Fig. 5. For ease of finding them, we have added the text in olive.

Comment 5: *While mathematically thorough, some derivations (e.g., in Section II-B) may be hard for readers without a strong background in differential equations and stochastic processes. Additional explanations are needed.*

Response: The authors thank the reviewer for the comment. We have added a paragraph at the beginning of Section II-B summarizing the goal and the main steps of the mathematical framework developed in this section. To highlight this additional paragraph, we have added the text in violet.

Reviewer 3's comments

Comment 1: *The paper acknowledges the importance of avoiding overfitting in inverse problem settings. It would be beneficial if the authors could discuss or experiment with standard regularization techniques commonly used in neural networks, such as dropout and batch normalization,. It would be interesting to assess whether these methods lead to notable improvements in prediction robustness and generalization, particularly under data noise or sparsity.*

Response: We thank the reviewer for the comment. We have run additional experiments with dropout introduced in the deterministic setting where we consider different values for the dropout rate (0.1 and 0.5), but they were either failing in removing the artifacts from the comparison or oversmoothing the approximation. This suggests that an uncertainty framework around the dynamics is more suited to reduce overfitting to irrelevant data that does not come from the dynamics. We have shown the results in Fig. 19 and Table I. For ease of exposition, we have highlighted the text in Section VI-D relevant to this comment in brown.

Comment 2: *In Figure 11(b), it appears that the neural network training has not yet converged. It is recommended that the authors extend the training for additional epochs to determine whether the loss curve eventually flattens. This could help confirm whether the observed trends are due to model capacity limitations or insufficient training duration.*

Response: The authors thank the reviewer for pointing this out. We have updated Fig. 15(b) with a greater number of epochs to show the convergence. The updated figure reveals that the plot in the previous version of the manuscript had insufficient training duration, which is corrected in this plot that shows clear convergence now. This figure conveys the message that there is a tradeoff between performance and memory cost. Higher number of shared layers reduces the memory cost, but the

performance gets inferior (giving higher error). For ease of exposition, we have highlighted the text in Section VI-C related to this comment in teal.

Comment 3: *The current numerical experiments do not include comparisons with other neural network-based approaches or classical inverse problem methods. Including such benchmarks would provide a more complete perspective on the advantages of the proposed framework. Additionally, given that neural networks are particularly well-suited to high-dimensional problems, it would strengthen the paper to include a higher-dimensional example to further illustrate the effectiveness and scalability of the proposed method.*

Response: We thank the reviewer for the comment. To address this, we have included some classical and neural network-based approaches in Section V-F. In addition, we did more experiments with some of these methods as baselines, and added qualitative and quantitative comparisons in VI-F.

Regarding the comment on higher dimensionality of the problem, we would like to emphasize that the goal of this paper is to address the end-to-end robust solutions for inverse problems in both deterministic as well as probabilistic settings, with an efficient balance between the performance and the compute (time) cost, and to provide a unified framework to do so for a class of polynomial dynamics. The paper does not focus on tackling very high-dimensional dynamics, primarily because such systems are not very common in electrical and power systems, which is the scope of this paper. Also, it is noteworthy that although neural networks are indeed efficient in learning nonlinear functions in high-dimensional parameter space, the main challenge that this paper is trying to address is the complexity of finding the solutions in inverse problems involving dynamics. In this context, it is more relevant to address the dimensionality of dynamics and not the parameter space. Hence, to address the reviewer's comment on higher dimensional problems, we have included an additional simplified benchmarking on an inverse problem for 2-dimensional (2-D) compressible Navier Stokes dynamics (also included this in the abstract of the paper). Similar equations are used to study the dynamical behavior of gas insulated circuit breakers. We have added the results in Section VI-B. For ease of exposition, we have added all additional texts in response to this comment in blue.

Additional remarks from the authors

The authors thank the reviewers for catching the typos. We have corrected the typos in the revised manuscript.

Additionally, for a better flow of the text, we made a few editorial changes in the paper. Also, we included a note on code availability in Section VI-H. We have highlighted them in magenta.

Authors' response to reviewer's comments

The authors thank the reviewers for their comments. In this document we address Reviewer 1’s comments in detail.

Reviewer 1’s comments

Comment 1: *The abstract should highlight specific quantitative outcomes (accuracy improvements, computational gains) rather than remaining general.*

Response: We thank the reviewer for the comment. We have added in the abstract a summary of the quantitative results that are compared with the baselines in blue.

Comment 2: *While several classical and neural approaches are mentioned, the quantitative comparison section could be expanded. Including explicit numerical performance tables (e.g., computation time, rates) would strengthen the validation.*

Response: We thank the reviewer for the comment. In the previous version of the paper, we added Tables I, III and IV, which capture quantitative results. In this version we expanded Table III. In particular, Table III now shows the benchmarking results of the performance (validation MSE) and training times of our proposed E2E methods along with a diverse set of baseline deterministic as well as probabilistic methods. Moreover, to further emphasize the key advantage of our proposed approach of achieving a balance between performance and cost, we compare the inference times with select baselines of deterministic and probabilistic approaches in Table IV. Additionally, we mentioned a qualitative comparison of the computation complexities in Table II. For ease of finding them, we have highlighted these in orange in Sections V-E and V-F.

Comment 3: *The discussion of practical usage should be more detailed.*

Response: We thank the reviewer for the comment. To address this, we have expanded the motivation in Section I-A. For ease of review, the text in the previous version of the manuscript relevant to this comment is highlighted in cyan (in Section I-A and Section V-B), which mentions several industrial monitoring applications that can benefit from the proposed approach and explains the set-up of a practical case study. The additional text in the current version of the manuscript further delving into practical applicability has been highlighted in violet.

Comment 4: *The mathematical derivations could be shortened or moved to an appendix to improve readability.*

Response: We thank the reviewer for the comment. In response, we shortened Section II-B by moving some of the mathematical derivations to Appendix A. For ease of exposition, we have highlighted the text in olive.

Comment 5: *Include a table with quantitative benchmarking results. Metrics such as training/inference time, validation, and model convergence speed compared to baseline methods would support claims of efficiency.*

Response: The authors thank the reviewer for the comment. In the previous version of the paper, we added Table IV to compare the computation times (training and inference) of the baseline probabilistic methods with our end-to-end probabilistic approach. In the current version we have expanded Table III to include the training times of all methods and modified Table IV to keep only inference times and include them for deterministic methods as well, to demonstrate the advantage of our proposed methods in both deterministic and probabilistic frameworks. Additionally, we updated Table II with more qualitative information that are relevant for computational cost. We note that these additional results are also relevant for Comment 2, and hence we highlight this additional text in orange in Section V-F, where we have included more details on each of the baselines and compare the results.

Comment 6: *The conclusion section could better emphasize the novelty and future potential.*

Response: We thank the reviewer for the comment. We have expanded Section VI, Conclusion to elaborate on some of the technical novelties as well as few examples of future applications of this research outcome. For ease of readability, we have highlighted the text in teal.

Comment 7: *Minor grammatical and stylistic edits must be considered.*

Response: The authors thank the reviewer for the comment. We have proofread the updated paper and included the grammatical as well as stylistic edits throughout the manuscript.

Comment 8: *Readability must be improved.*

Response: The authors thank the reviewer for the comment. To facilitate better readability, we have added Section I-E summarizing the organization of the paper, added an introductory paragraph in Sections II-B and V-F. Also, we restructured Section IV and the text of Section V-F for a better flow of readability. For ease of reference, we have highlighted the added text in magenta. Also, following reviewer's comment 4, we have moved the mathematical derivations to the appendix, which we hope will also improve the readability.

Summary:

This paper presents an end-to-end framework for robust inverse problem-solving in electrical dynamical systems using Physics-Informed Neural Network (PINN). The authors introduce a probabilistic extension of Physics-Informed Neural Networks (Pro-PINN) that provides both point estimates and uncertainty quantification for inferred variables, enabling confidence-aware predictions even under sparse and noisy data. Key methodological contributions include a theoretical framework for uncertainty propagation, a stability-aware loss weighting scheme to mitigate singularities, a history-aware training strategy to address temporal error accumulation, and an active sampling algorithm for efficient sensor placement. The proposed methods, validated on some numerical examples can accurately predict heat distribution beyond current physics simulation capabilities.

Comments:

1. The paper acknowledges the importance of avoiding overfitting in inverse problem settings. It would be beneficial if the authors could discuss or experiment with standard regularization techniques commonly used in neural networks, such as dropout and batch normalization,. It would be interesting to assess whether these methods lead to notable improvements in prediction robustness and generalization, particularly under data noise or sparsity.

2. In Figure 11(b), it appears that the neural network training has not yet converged. It is recommended that the authors extend the training for additional epochs to determine whether the loss curve eventually flattens. This could help confirm whether the observed trends are due to model capacity limitations or insufficient training duration.

3. The current numerical experiments do not include comparisons with other neural network-based approaches or classical inverse problem methods. Including such benchmarks would provide a more complete perspective on the advantages of the proposed framework. Additionally, given that neural networks are particularly well-suited to high-dimensional problems, it would strengthen the paper to include a higher-dimensional example to further illustrate the effectiveness and scalability of the proposed method.

Typos:

The paper in general is well-written and organized. Here are some small typos.

Page2 left B: “a type pf SciML” to “a type of SciML”…

Page9 right VI A: “endure fidelity in the approximation” to “ensure high accuracy in the approximation”
