# Supplementary material for: End-to-end robust system discovery in electrical dynamical systems using scientific machine learning
Source: Commun Eng. 2026 Jun 16;5:172. doi: 10.1038/s44172-026-00689-2 (PMC13645738; doi:10.1038/s44172-026-00689-2)
Supplement: Supplementary file 2 [file 44172_2026_689_MOESM2_ESM.pdf]

Supplementary Document for *End-to-End Robust System Discovery in Electrical Dynamical Systems using Scientific Machine Learning*

Jhelum Chakravorty, Nicolò Ripamonti and Tor Laneryd

Uncertainty Quantification in Polynomial Dynamics and Singularity Avoidance in Inverse Problems

Let $\mathcal{C}$ and $\mathcal{E}$ be connected by the following dynamics.

$$\mathcal{E} = \mathcal{D}(\mathcal{C}), \tag{1}$$

where $\mathcal{D}$ a polynomial diffeomorphism (e.g., in partial differential form). We assume that it does not vanish identically.

In coherence with the discussion so far, we restrict our study within the lieu of the following assumption:

A1 The effect is a spatiotemporal polynomial function that can be expressed as the solution of a polynomial differential equation.

We consider the following two instances of the cause-effect relationship:

a) Additive relationship: In this case $\mathcal{D}$ takes the form:

$$\frac{\partial \mathcal{E}(x,t)}{\partial t} + \sum_{i=0}^{m_1} \alpha_i \nabla_x^i \mathcal{E}(x,t) + \sum_{j=0}^{m_2} \beta_j \mathcal{E}^j(x,t) = \gamma \mathcal{C}(x,t), \tag{2}$$

where coefficients $\alpha_i, \beta_j, \gamma, m_1, m_2 > 0$ are known and fixed and at least one of α_i and β_j is positive, and ∇^i is the i-th order differential operator.

For making (2) non-trivial, we assume the following.

A2 The terms $\mathcal{S}_1 := \sum_{i=0}^{m_1} \alpha_i \nabla_x^i \mathcal{E}(x,t)$ and $\mathcal{S}_2 := \sum_{j=0}^{m_2} \beta_j \mathcal{E}^j(x,t)$ are not equal, and none of these terms is identically zero.

In this case it is straightforward to see that the additive inverse $\mathcal{C}$ exists and is given by

$$\mathcal{C}(x,t) = \frac{1}{\gamma}\left(\frac{\partial \mathcal{E}(x,t)}{\partial t} + \sum_{i=0}^{m_1} \alpha_i \nabla_x^i \mathcal{E}(x,t) + \sum_{j=0}^{m_2} \beta_j \mathcal{E}^j(x,t)\right) \tag{3}$$

b) Multiplicative relationship: In this case $\mathcal{D}$ takes one of the following two forms:

$$\frac{\partial \mathcal{E}(x,t)}{\partial t} + \Big[\sum_{i=0}^{m_1} \alpha_i \nabla_x^i \mathcal{E}(x,t)\Big]\mathcal{C}(x,t) + \sum_{j=0}^{m_2} \beta_j \mathcal{E}^j(x,t) = 0, \tag{4a}$$

and,

$$\frac{\partial \mathcal{E}(x,t)}{\partial t} + \sum_{i=0}^{m_1} \alpha_i \nabla_x^i \mathcal{E}(x,t) + \Big[\sum_{j=0}^{m_2} \beta_j \mathcal{E}^j(x,t)\Big]\mathcal{C}(x,t) = 0, \tag{4b}$$

Combining the above two, we get a third multiplicative form

$$\frac{\partial \mathcal{E}(x,t)}{\partial t} + \Big[\sum_{i=0}^{m_1} \alpha_i \nabla_x^i \mathcal{E}(x,t) + \sum_{j=0}^{m_2} \beta_j \mathcal{E}^j(x,t)\Big]\mathcal{C}(x,t) = 0. \tag{4c}$$

Since (4c) can be derived from (4a) and (4b), we leave it out from the discussion here.

Lemma 1. *Under the assumptions A1 and A2, for the multiplicative relationship, $\mathcal{C}$ can be expressed as the solution of a polynomial differential equation iff (if and only if) $\mathcal{S}_1 = 1$ and $\mathcal{S}_2 = 1$.*

Proof. We prove the lemma for $\mathcal{S}_1 = 1$. The proof for $\mathcal{S}_2 = 1$ follows using similar arguments.

If: we have $\mathcal{S}_1 = 1$. Since $\mathcal{C}(x,t)$ can be expressed as:

$$\mathcal{C}(x,t) = -\frac{\partial \mathcal{E}(x,t)}{\partial t}\mathcal{S}_1^{-1} - \sum_{j=0}^{m_2} \beta_j \mathcal{E}^j(x,t)\mathcal{S}_1^{-1}, \tag{5}$$

we have

$$\mathcal{C}(x,t) = -\frac{\partial \mathcal{E}(x,t)}{\partial t} - \sum_{j=0}^{m_2} \beta_j \mathcal{E}^j(x,t),$$

which is the solution of a polynomial differential equation.

Only if: we assume that $\mathcal{C}$ is a polynomial differential equation. Then, we prove this by contradiction. Since $\mathcal{S}_1$ is a polynomial, its multiplicative inverse is a $\mathcal{S}_1^{-1}$ is a rational fraction. Since $\mathcal{S}_1$ is not equal to 1, $\mathcal{S}_1^{-1}$ is a rational fraction that cannot be expressed as a polynomial (since its denominator is not equal to 1). Then the proof follows directly from the fact that multiplication of two polynomials is a polynomial and that the addition of two polynomials is a polynomial. From (5) it follows that the first term is not a polynomial and the second term is also not a polynomial due to assumption (A2). Then, by construction $\mathcal{C}(x,t)$ is the solution of a rational fraction differential equation which can't be expressed with polynomial terms, which contradicts assumption (A1). Hence, by contradiction, we have that $\mathcal{S}_1 = 1$. □

It can be inferred from Lemma 1 that the inverse solution is usually a rational fraction (and not a polynomial) when the cause and effects are in multiplicative relation. Some of

Jhelum Chakravorty is with Hitachi Energy Research Canada, Montreal, Canada (e-mail: jhelum.chakravorty@hitachienergy.com).

Nicolò Ripamonti is with Hitachi Energy Research Switzerland, Baden-Dätwil, Switzerland (e-mail: nicolo.ripamonti@hitachienergy.com).

Tor Laneryd is with Hitachi Energy Research Sweden,Västerås, Sweden (e-mail: tor.laneryd@hitachienergy.com).

these rational fractions could have singularities, making the inverse solution ill-posed. We discuss one way of avoiding such singularities to solve the inverse problems.

Impact of Gaussian Uncertainty of the Effect on the Cause

An important aspect of uncertainty quantification of cause in inverse problems is to quantify the impact of uncertainty in the effect on the cause. In this section we derive an analytical expression of the Gaussian uncertainty applied to the effect in the systems defined by (2)–(4b).

Let $\mathcal{E} \sim \mathcal{N}(\mu_{\mathcal{E}}, \Sigma^2_{\mathcal{E}})$. Then, it is straightforward to show that $\mathcal{E}(x,t) = \mu_{\mathcal{E}} + \Sigma^2_{\mathcal{E}} Z$, where $Z \sim \mathcal{N}(0,1)$. Then, (2) can be expanded as

$$\mathcal{C}(x,t) = \frac{1}{\gamma}\Big[\partial\mathcal{E} + \sum_{i=0}^{m_1} \alpha_i \nabla^i \mathcal{E} + \sum_{j=0}^{m_2} \beta_j \mathbb{A}_j\Big], \tag{6a}$$

where

$$\partial\mathcal{E} := \frac{\partial \mu_{\mathcal{E}}}{\partial t} + \frac{\partial \Sigma^2_{\mathcal{E}}}{\partial t} Z \sim \mathcal{N}\Big(\frac{\partial \mu_{\mathcal{E}}}{\partial t}, \frac{\partial \Sigma^2_{\mathcal{E}}}{\partial t}\Big), \tag{6b}$$

$$\nabla^i\mathcal{E} := \nabla^i \mu_{\mathcal{E}} + \nabla^i \Sigma^2_{\mathcal{E}} Z \sim \mathcal{N}\big(\nabla^i \mu_{\mathcal{E}}, \nabla^i \Sigma^2_{\mathcal{E}}\big), \tag{6c}$$

$$\mathbb{A}_j := (\mu_{\mathcal{E}} + \Sigma^2_{\mathcal{E}} Z)^j. \tag{6d}$$

Since higher order polynomials of a standard normal variable are not usually normally distributed, one may estimate $\mathbb{A}_j$ using parametric method such as deep neural network (DNN) or non-parametric method such as Kernel Density Function (KDE) or Gaussian Mixture Models (GMM). Alternatively, Polynomial Chaos Expansion[1] with Hermite polynomials can be employed to represent $\mathbb{A}_j$ as a series expansion, leveraging the natural orthogonality properties of Hermite polynomials with respect to Gaussian measures. Hence, the schematic for estimating the inverse solution $\mathcal{C}$ of an effect $\mathcal{E}$ with a Gaussian uncertainty can be summarized in Fig. 1, where $\mathcal{F}$ denotes the parametric or non-parametric model for estimating the $\mathbb{A}_j$ in (6d).

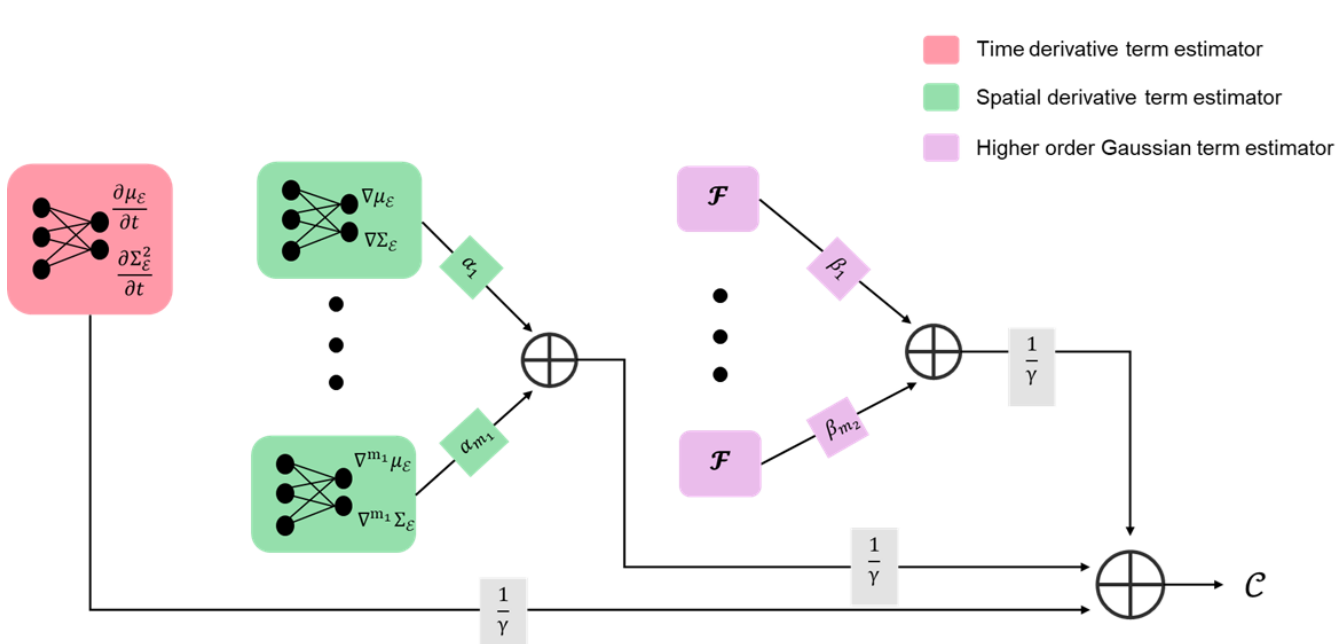

Fig. 1: Schematic diagram for end-to-end estimation of multiplicative inverse solution for effects endowed with Gaussian uncertainty.

Singularity Avoidance in Inverse Problems

In inverse problems for electrical dynamical systems, singularities arise when the mapping from effect $\mathcal{E}$ to cause $\mathcal{C}$ becomes ill-posed or numerically unstable, essentially violating assumption A2. For our heat diffusion problem, singularities typically occur at points where the sensitivity of the PDE residual approaches zero, making the inversion process highly unstable. We propose a strategy for singularity avoidance based on the local stability of the inverse problem. Let us define a stability metric $\eta(x,t)$ at each collocation point $(x,t) \in \Omega$ as:

$$\eta(x,t) = \left|\frac{\partial F[\mathcal{E};\mathcal{C}]}{\partial \mathcal{E}}\right| \tag{7}$$

where $F[\mathcal{E};\mathcal{C}]$ is the PDE residual of predicting the dynamics, denoted with a slight abuse of notation for the sake of simplicity. Small values of $\eta(x,t)$ indicate regions where small changes in temperature can lead to disproportionately large changes in estimated heat loss. Our approach employs a continuous weighting function $w(\eta)$ that diminishes the influence of potentially singular points during training:

$$w(\eta) = \frac{\eta^2}{\eta^2 + \epsilon^2} \tag{8}$$

where ϵ is a small threshold parameter. This modified weight is incorporated into the loss function (2) to yield:

$$\mathcal{L}(\theta;\Omega) = w(\eta(x,t))\omega_1 L_1(\theta;x,t) + \omega_2 L_2(\theta;x,t) \tag{9}$$

The sample exclusion approach can be viewed as a special case of this algorithm where $w(\eta)$ is a step function:

$$w(\eta) = \begin{cases} 0, \text{if } \eta < \epsilon \\ 1, \text{otherwise} \end{cases} \tag{10}$$

This binary weighting removes near-singular points from the training process entirely, offering a simpler implementation at the cost of potentially steep transitions in the training domain. In practice, we implement this approach using an iterative process during training:

1) At each optimization step, compute the stability metric $\eta(x,t)$ at all collocation points in the current batch
2) Apply the weighting function $w(\eta)$ to adjust the contribution of each point to the loss
3) Update model parameters through gradient descent on the weighted loss

This approach ensures numerical stability throughout the training process while preserving valuable information from stable regions, leading to more robust heat loss estimates in our electrical device applications.

Benchmarking of UQ in Nonlinear Dynamics with Burgers Equation

To demonstrate the effectiveness of our proposed PIML approach for inverse problems, we first consider the one-dimensional Burgers equation as an introductory benchmark case. This PDE is an instance of a second-order polynomial differential equation with multiplication relationship between the effect $\mathcal{E}$ being the viscous field u and the cause $\mathcal{C}$ being the diffusion coefficient μ. It defines multiple dynamical phenomena in electrical and power devices such as in fluid mechanics, nonlinear acoustics and gas dynamics and serves as an ideal test-bed due to its nonlinear characteristics and well-understood analytical properties, while being computationally tractable for comprehensive validation. We consider

the viscous Burgers equation with spatially varying diffusion coefficient:

$$\frac{\partial u}{\partial t} + u\frac{\partial u}{\partial x} = \mu(x)\frac{\partial^2 u}{\partial x^2} \tag{11}$$

defined on the spatial domain $x \in [0, 1]$ with periodic boundary conditions and initial condition:

$$u(x, 0) = -\sin(\pi x). \tag{12}$$

The inverse problem consists of identifying the spatially varying diffusion parameter $\mu(x)$ from observations of the solution field $u(x, t)$. For this study, we define the true diffusion parameter as:

$$\mu(x) = \frac{(\arctan(100x) + \pi/2)}{\pi} \cdot \frac{0.2}{\pi} + \frac{0.01}{\pi} \tag{13}$$

This parameterization creates a smooth transition from low to high diffusion values, providing a challenging test case that exhibits both gradual and steep spatial variations in the parameter field. Our PIML approach follows the framework outlined in Methodology for PIML section of the main paper, with the following detailed implementation strategy:

- We generate synthetic measurement data by solving the forward problem using a high-resolution finite difference scheme with 512 spatial points and RK4 for time integration to ensure high accuracy in the approximation.
- We implement two separate neural networks within our Pro-PINN framework: one for approximating the solution field $\hat{u}(x, t; \theta_u)$ and another for the spatially varying diffusion parameter $\hat{\mu}(x; \theta_\mu)$. Each network consists of 8 hidden layers with 64 neurons per layer, utilizing Mish activation function to ensure the differentiability required for automatic differentiation. The solution network takes spatiotemporal coordinates (x, t) as input, while the parameter network depends only on spatial coordinate x. Both networks output mean and variance estimates in the probabilistic setting, effectively doubling the output dimensions to accommodate uncertainty quantification.
- Following the theoretical framework developed in Uncertainty quantification problem section of the main paper and Uncertainty Quantification in Polynomial Dynamics and Singularity Avoidance in Inverse Problems section of this supplementary document, we address the challenge that higher-order polynomials of Gaussian random variables are not necessarily Gaussian-distributed. We employ Polynomial Chaos Expansion with Hermite polynomials to analytically capture the uncertainty propagation. Since the state and its partial derivative are modeled as Gaussian random variables, their product can be approximated using bivariate Hermite polynomial expansion with maximum polynomial order of $P = 4$.
- We implement the stability-based weighting approach described in Singularity Avoidance in Inverse Problems section, computing the stability metric at all collocation points and establishing a threshold $\epsilon = 10^{-6}$ based on empirical analysis of the stability metric distribution.

Fig. 2 presents a comprehensive view of the Pro-PINN performance, showing the exact solution $u(x, t)$, the network approximation, the absolute error, and the predicted uncertainty map. The results demonstrate that our approach successfully captures the complex spatiotemporal dynamics of the Burgers equation, with errors remaining well within the predicted uncertainty bounds throughout most of the domain. Notably, higher uncertainty regions correspond to areas where sharp gradients dominates, indicating that the model appropriately identifies regions of increased prediction difficulty. Fig. 3 provides a temporal analysis by comparing

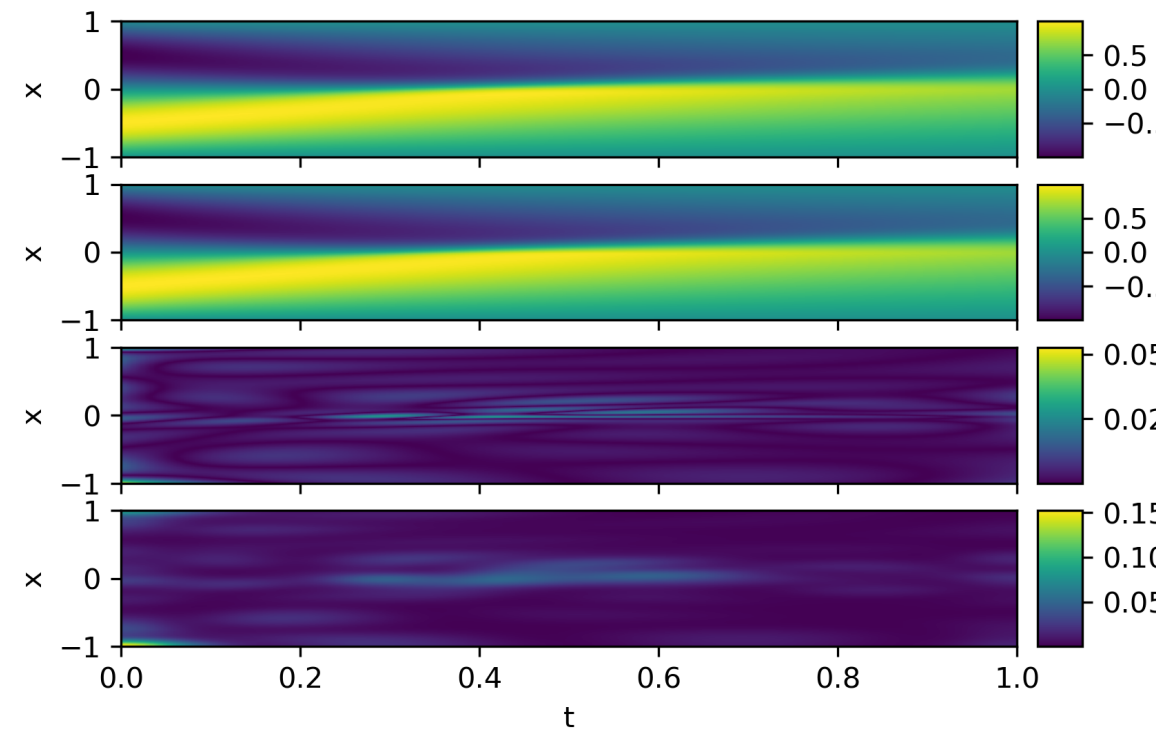

Fig. 2: The figure shows (from top to bottom row) the exact solution $u(x, t)$, Pro-PINN approximation $\hat{u}(x, t)$, absolute error $|u - \hat{u}|$, and predicted uncertainty $u(x, t)\sigma_u(x, t)$. The uncertainty field correlates well with error distribution, with highest values in shock regions and steep gradient areas.

error slices at different time instances against three times the predicted standard deviation. This visualization confirms that the uncertainty quantification is well-calibrated and the error is consistent with Gaussian uncertainty bounds. The temporal evolution shows that uncertainty increases in regions where shock-like features develop, demonstrating the model's ability to adapt its confidence based on local solution characteristics.

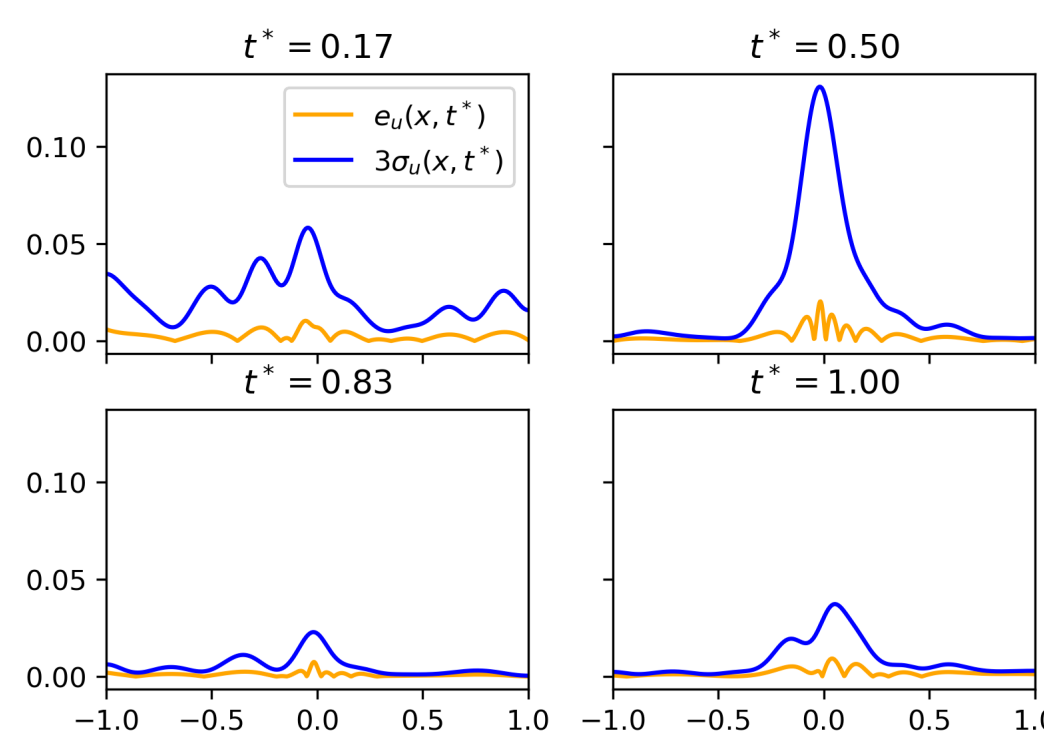

Fig. 3: Temporal validation of uncertainty calibration. Error slices $e_u(x, t^*) := |u(x, t^*) - \hat{u}(x, t^*)|$ (orange lines) at 4 time instances compared against $3\sigma_u$ confidence prediction. Errors consistently fall below predicted uncertainty, demonstrating excellent uncertainty calibration and spatially-adaptive confidence estimates.

The most critical validation of our inverse problem approach is presented in the parameter identification results. The comparison between the true diffusion parameter $\mu(x)$ and its

Pro-PINN approximation, shown in Fig. 4, reveals excellent agreement across the spatial domain. The predicted uncertainty bands appropriately widen in regions where the parameter exhibits rapid spatial variation (around $x \approx 0$), indicating that the model correctly identifies areas of increased estimation difficulty, even for the parameter of interest.

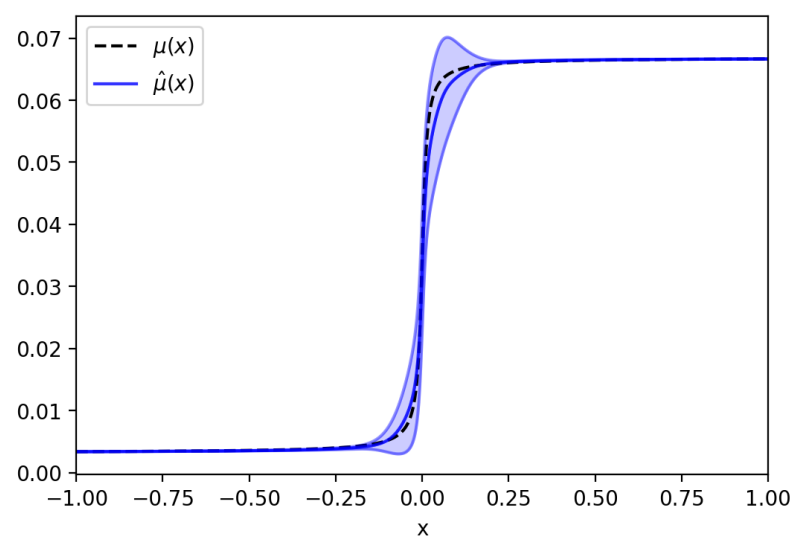

Fig. 4: Inverse identification of spatially varying diffusion parameter. True parameter $\mu(x)$ (black line) versus Pro-PINN estimate $\hat{\mu}(x)$ (blue line) with $3\sigma_\mu(x)$ uncertainty bands (shaded region). The network accurately reconstructs both smooth and sharp transition regions, with uncertainty bands widening appropriately in challenging areas.

These results validate several key aspects of our methodology, including the effectiveness of uncertainty quantification in providing reliable confidence estimates, and the robustness of the approach in handling challenging nonlinear dynamics. The successful parameter identification demonstrates the power of physics-informed constraints in regularizing the inverse problem, while the well-calibrated uncertainty estimates provide the confidence metrics essential for practical deployment.

Benchmarking of UQ in 2-D Nonlinear Dynamics with Compressible Navier Stokes Equations

To further evaluate the capabilities of our proposed framework on a more complex physical system, we now consider the two-dimensional compressible Navier-Stokes (CNS) equations. This system is significantly more challenging than the Burgers' equation due to the coupling between multiple state variables (density, momentum, and energy) and the presence of more complex nonlinearities. The CNS equations are fundamental in describing the motion of viscous, compressible fluids and appear in numerous scientific and engineering applications, from aerodynamics to astrophysics.

In this benchmark, we frame the problem as an inverse problem: we aim to identify unknown, spatially varying body forces, $f_x(x, y)$ and $f_y(x, y)$, using measurements of the flow field variables. This type of inverse problem is highly relevant in fields where external forces acting on a fluid are difficult or impossible to measure directly. For example, in magnetohydrodynamics, these forces could represent electromagnetic fields acting on a plasma, or in geophysical fluid dynamics, they might represent effects of unresolved physical processes. Successfully inferring these force fields from observable data demonstrates the robustness and practical utility of our PIML-based approach for complex, multi-physics systems.

The governing equations for this problem are given by:

$$\frac{\partial Q}{\partial t} + \frac{\partial F}{\partial x} + \frac{\partial G}{\partial y} = \frac{\partial H}{\partial x} + \frac{\partial J}{\partial y} + S \tag{14}$$

where Q is the vector of conserved variables, F and G are the inviscid flux vectors, H and J are the viscous flux vectors, and S is the source term vector due to body forces. The source term we seek to identify is:

$$S = \begin{bmatrix} 0 \\ f_x \\ f_y \\ uf_x + vf_y \end{bmatrix} \tag{15}$$

The term $uf_x + vf_y$ in the energy equation represents the rate of work done by the body forces on the fluid. For this study, we define the true forcing functions as a stationary, spatially varying field designed to generate a non-trivial flow pattern:

$$f_x(x, y) = 10\sin(\pi x) \tag{16}$$
$$f_y(x, y) = 10\sin(\pi y) \tag{17}$$

The objective of the Pro-PINN model is to reconstruct the functions $f_x(x, y)$ and $f_y(x, y)$ from sparse and potentially noisy measurements of the velocity (u, v), density (ρ), and pressure (P) fields. Our PIML approach for this problem involves the following implementation strategy:

- We generate synthetic measurement data by solving the forward problem using a high-resolution finite-volume scheme on a 200×200 staggered grid over the spatial domain $(x, y) \in [0, 2\pi] \times [0, 2\pi]$. The simulation is run for a total of 140 time steps over an interval of [0, 0.0084]s, using a fourth-order Runge-Kutta time integrator, which ensures high accuracy in the ground truth solution.
- We implement two separate neural networks within the Pro-PINN framework: one for approximating the solution field $\hat{Q}(x, y, t; \theta_Q)$ and another for the spatially varying forcing terms $(\hat{f}_x(x, y; \theta_f), \hat{f}_y(x, y; \theta_f))$. Each network consists of 8 hidden layers with 256 neurons per layer, utilizing the Mish activation function. The solution network takes spatiotemporal coordinates (x, y, t) as input, while the forcing term network depends only on spatial coordinates (x, y). Both networks output mean and variance estimates to facilitate uncertainty quantification.
- Following the theoretical framework from Section II-B, we address the non-Gaussian nature of the propagated uncertainty through the nonlinear terms in the CNS equations (e.g., products of state variables). We employ Polynomial Chaos Expansion with Hermite polynomials to analytically capture the resulting distributions.
- We implement the stability-based weighting approach described in Section II-B2 to regularize the inverse problem. The stability metric is computed at all collocation points, and a threshold of $\epsilon = 10^{-6}$ is established based on empirical analysis to down-weight regions where the inversion is potentially unstable.

The results of our Pro-PINN framework on this challenging benchmark are summarized in Figures 5, 6, and 7. A qualitative assessment of the model's performance is presented

in Fig. 5, which visualizes the pressure field. The network's prediction closely matches the exact solution, demonstrating that the model can successfully capture the complex, coupled dynamics of the compressible flow. More importantly, the predicted uncertainty map shows a strong visual correlation with the absolute error map. This indicates that the model correctly identifies regions of higher difficulty, specifically areas with strong pressure gradients, and assigns greater uncertainty to them, a key feature for building trust in its predictions.

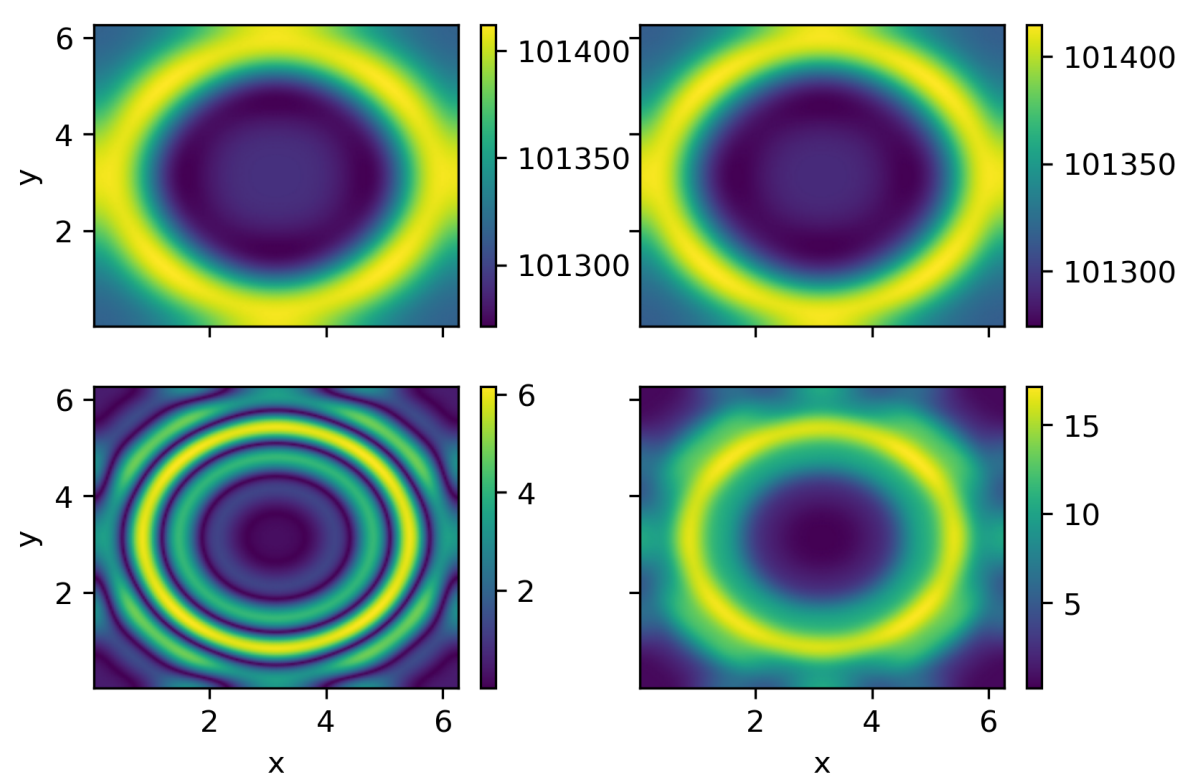

Fig. 5: The figure shows (in the clockwise order from top left): the exact pressure field $P(x, y, t)$, the Pro-PINN approximation $\hat{P}(x, y, t)$, the predicted uncertainty map $\sigma_P(x, y, t)$, and the absolute error $e_u(x, y, t) := |P(x, y, t) - \hat{P}(x, y, t)|$. The uncertainty field correlates well with the error distribution, with the highest values appearing in regions with strong pressure gradients and complex flow structures.

To verify this observation quantitatively, Fig. 6 provides a more rigorous validation of the model's uncertainty estimates through spatial cross-sections. The top row confirms the excellent agreement between the predicted and exact pressure profiles. The bottom row is critical, as it directly compares the actual prediction error against the model's self-reported 3σ confidence interval. The error consistently remains within these bounds, confirming that the uncertainty is well-calibrated. This means the model's confidence in its predictions is reliable and adapts appropriately across different regions of the domain.

Finally, Fig. 7 presents the solution to the core inverse problem: the identification of the unknown body forces, f_x and f_y. The plots show a remarkable agreement between the network's reconstruction and the true forcing functions. The model accurately recovers the sinusoidal shape and magnitude of the forces using only sparse measurements of the flow field. The narrowness of the uncertainty bands signifies high confidence in the inferred parameters. Together, these results validate that the physics-informed framework can effectively regularize and solve a complex, multi-physics inverse problem while providing reliable metrics of uncertainty.

Additional Results

This section provides more details and results to further discuss select topics in the main paper.

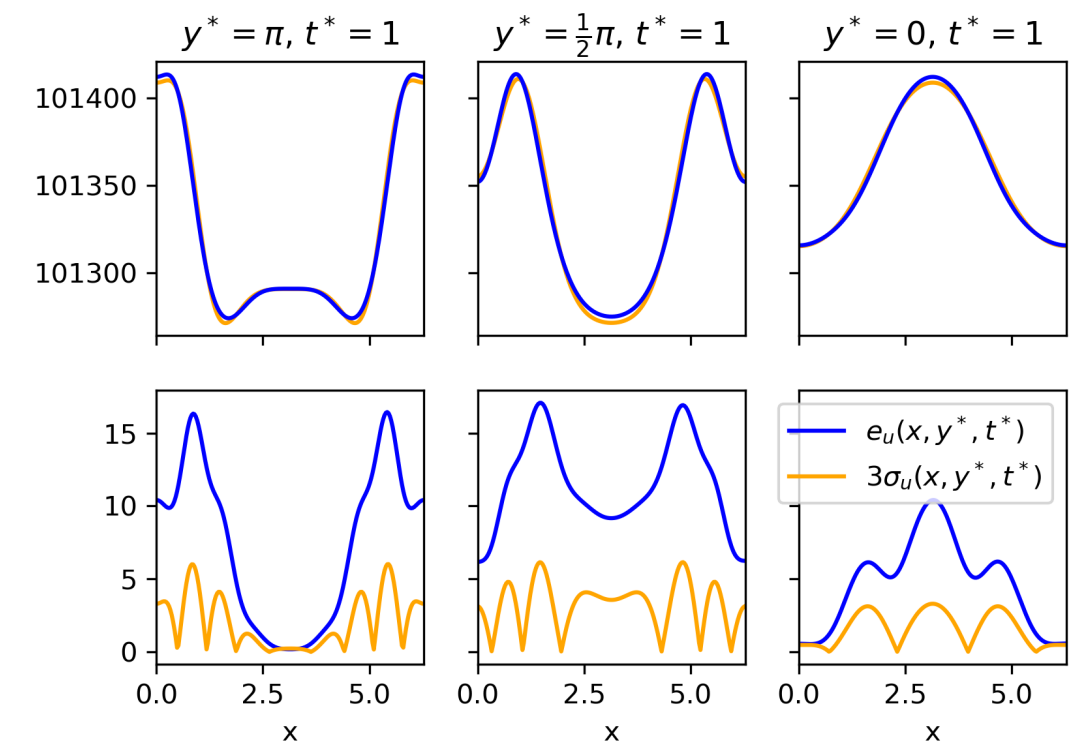

Fig. 6: Temporal validation of uncertainty calibration. In the top row, the exact solution is compared with its Pro-PINN approximation at three spatial cross-sections. In the bottom row, the corresponding absolute error e_u is compared with the $3\sigma_u$ uncertainty band. Errors consistently fall below the predicted uncertainty, demonstrating excellent uncertainty calibration and providing reliable, spatially-adaptive confidence estimates.

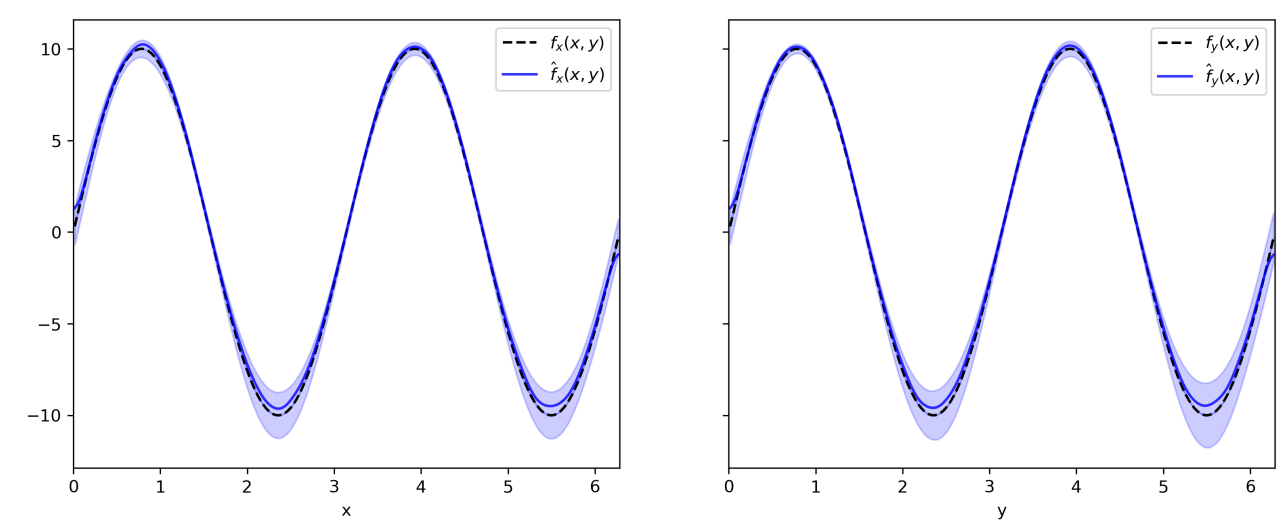

Fig. 7: Inverse identification of the spatially varying body forces. True parameters $f_x(x, y)$ and $f_y(x, y)$ (black dashed lines) versus Pro-PINN estimates $\hat{f}_x(x, y)$ and $\hat{f}_y(x, y)$(blue lines) uncertainty bands. The network accurately reconstructs the underlying forcing functions, with appropriately narrow uncertainty bands that validate the framework's effectiveness for complex inverse problems.

Active sampling algorithm

Algorithm 1 describes the active sampling approach for improving performance of the proposed method in presence of data sparsity explained in Active-sampling and robustness with data sparsity section of the main paper.

Qualitative Comparisons of Deterministic, Deterministic with Dropouts and Probabilistic PINNs

Fig. 8 shows the comparative performances of deterministic model with and without dropouts and with two variants (semi and full) of Pro-PINNs in estimating the temperature (effect in thermal diffusion process) and heat loss (cause in thermal diffusion process). For the deterministic case, we consider the effect of dropout regularization, with two different dropout rates (0.1 and 0.5), to show the impact of dropout in the performance. The rightmost column of Fig. 8b shows the ground truth measurements of temperature, which is missing from Fig. 8a since ground truths are not available for the

Algorithm 1 Active-Sampling algorithm for PINNs

Define set of distance thresholds $D_p := \{d_p^1, \dots, d_p^k\}$.
Define initial set of samples in space $\Omega_{\hat{\mathbf{x}}} = \{\hat{\mathbf{x}}_1, \hat{\mathbf{x}}_2, \dots, \hat{\mathbf{x}}_{m_{AC}}\}$ with $m_{AC} \ll |\Omega_{\mathbf{x}}|$
Train PINN model $g(\mathbf{x}, t; \theta)$ on $\Omega_{\hat{\mathbf{x}}}$.
for iterations $= 1, 2, \dots, n$, with n number of sampling iterations **do**
  for $d_p^i = d_p^1, d_p^2, \dots, d_p^k$ **do**
    Let $g_i(\mathbf{x}, t; \theta) = g(\mathbf{x}, t; \theta)$.
    Find $\Omega_{\mathbf{s}}^i \subset \Omega_{\mathbf{x}}$, with $|\Omega_{\mathbf{s}}^i| = m_{AC}$, s.t.

$$\max_{\mathbf{x} \in \Omega_{\mathbf{s}}^i} \|\hat{T} - T\|_2^2 \tag{18}$$

    with $dist(x_i, \Omega_{\hat{\mathbf{x}}} \cup \Omega_{\mathbf{s}}^i \setminus x_i) > d_p^i$ and $dist(x_i, \partial\Omega_{\mathbf{x}}) > d_b$
  end for
  Train PINN model $g_i(\mathbf{x}, t; \theta)$ on $\Omega_{\hat{\mathbf{x}}} := \Omega_{\mathbf{s}}^* \cup \Omega_{\hat{\mathbf{x}}}$ with $\Omega_{\mathbf{s}}^*$ such that $\max_{\mathbf{x} \in \Omega_{\hat{\mathbf{x}}}^*} \|\hat{T} - T\|_2^2 \geq \max_{\mathbf{x} \in \Omega_{\hat{\mathbf{x}}}^i} \|\hat{T} - T\|_2^2$
  Let $\Omega_{\hat{\mathbf{x}}} = \Omega_{\hat{\mathbf{x}}}^i$ and $g(\mathbf{x}, t; \theta) = g_i(\mathbf{x}, t; \theta)$.
end for
Return $\Omega_{\hat{\mathbf{x}}}$.

inverse problems. We can see therein that the deterministic models predict the artifacts that are not part of thermal diffusion process (such as the cables dangling on the wall) due to the overfitting to data. This effect is reduced in the semi Pro-PINN and almost absent in the full Pro-PINN. The effect of overfitting can be reduced to the deterministic PINN through a regularization term but needs a manual inspection through hyper-parameter tuning, whereas this is achieved in a more automated way in Pro-PINN estimates. We also evaluate dropout as a form of regularization.

References

[1] B. Sudret, "Global sensitivity analysis using polynomial chaos expansions," *Reliability engineering & system safety*, vol. 93, no. 7, pp. 964–979, 2008.

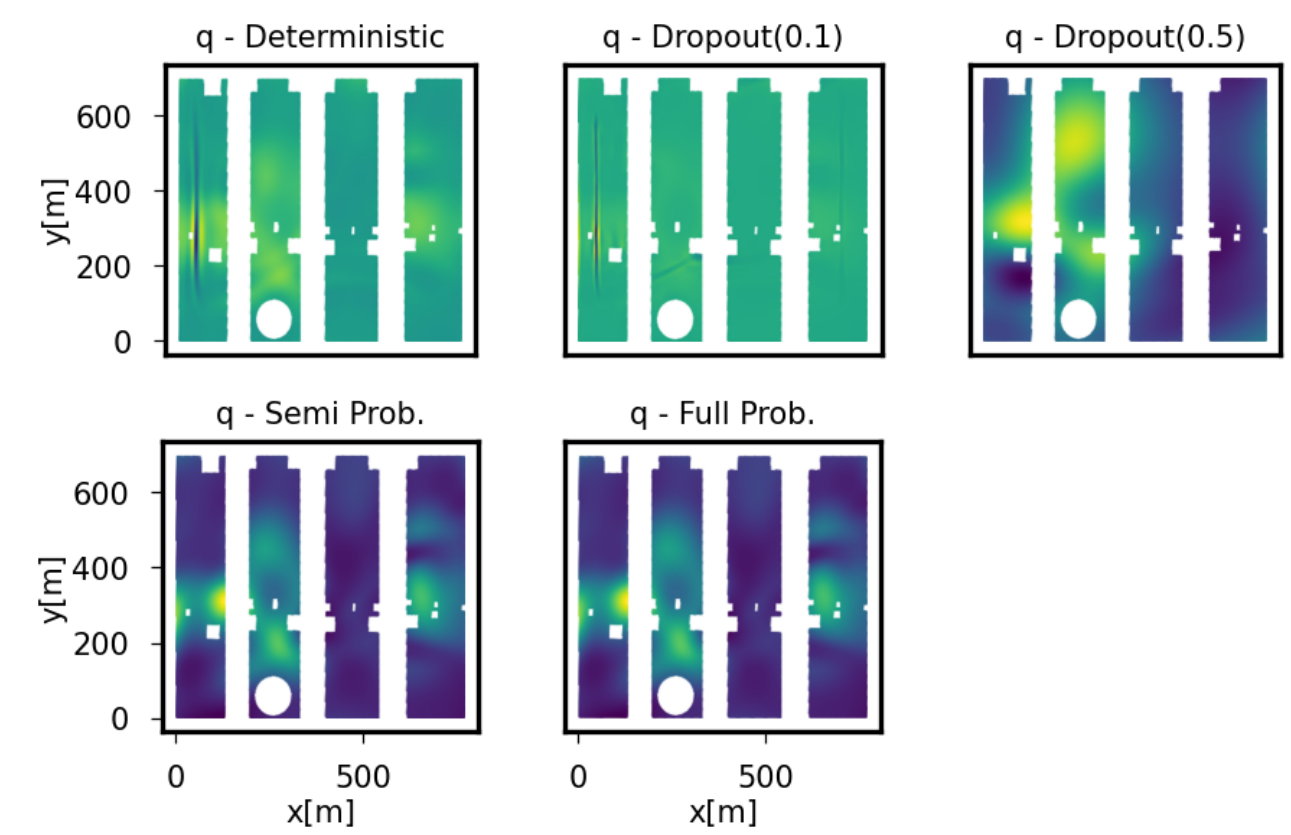

(a)

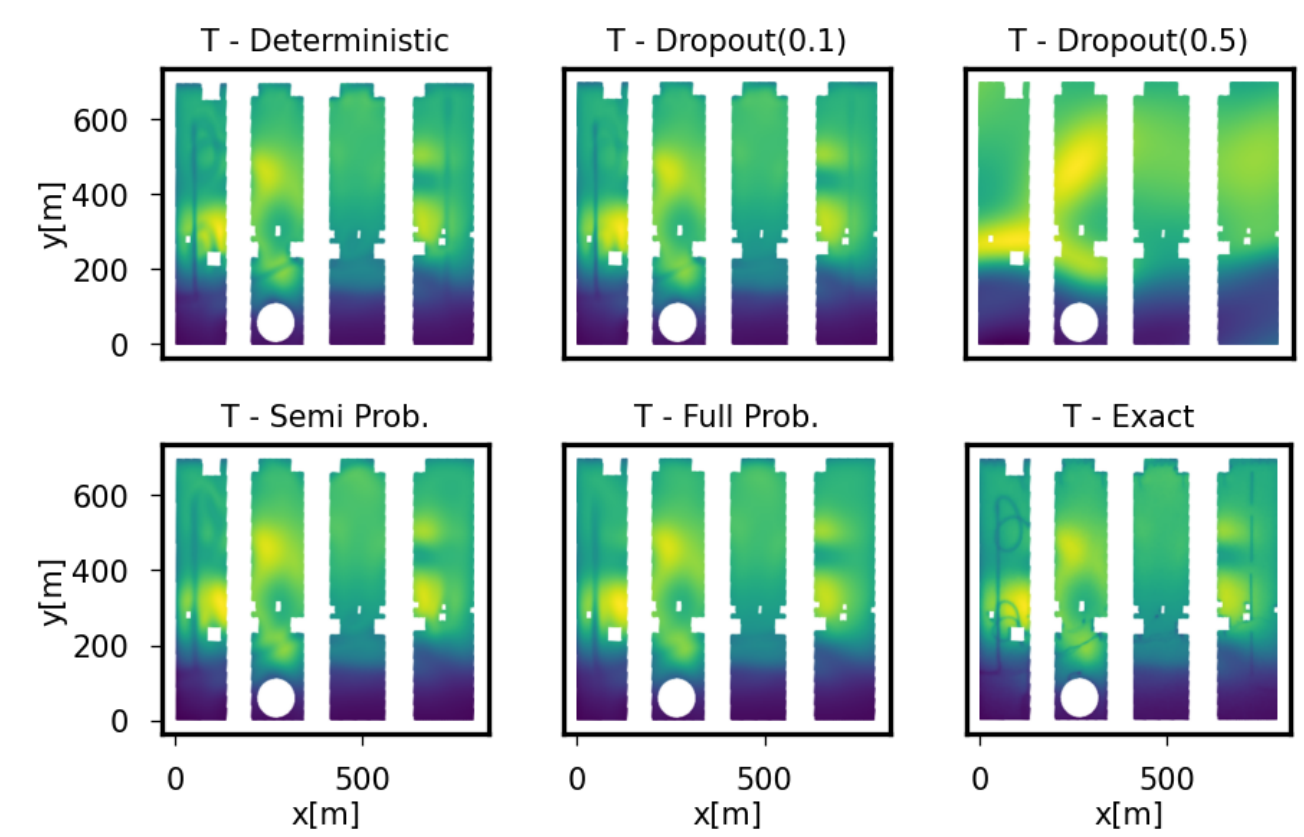

(b)

Fig. 8: Comparison of deterministic and probabilistic PINNs' predictions at $t = 285s$. (a) Local heat loss distribution. (b) Temperature distribution.
